# Analytical Methods for Chemical and Sensory Characterization of Scent-Markings in Large Wild Mammals: A Review

**DOI:** 10.3390/s140304428

**Published:** 2014-03-05

**Authors:** Simone B. Soso, Jacek A. Koziel, Anna Johnson, Young Jin Lee, W. Sue Fairbanks

**Affiliations:** 1 Environmental Science Interdepartmental Graduate Program, Iowa State University, Ames, IA 50011, USA; E-Mail: sbsoso@iastate.edu; 2 Department of Agricultural and Biosystems Engineering, Iowa State University, Ames, IA 50011, USA; 3 Department of Animal Science, Iowa State University, Ames, IA 50011, USA; E-Mail: johnsona@iastate.edu; 4 Department of Chemistry, Iowa State University, Ames, IA 50011, USA; E-Mail: yjlee@iastate.edu; 5 Department of Natural Resource Ecology and Management, Oklahoma State University, Stillwater, OK 74078, USA; E-Mail: sue.fairbanks@okstate.edu

**Keywords:** scent-marking, semiochemicals, pheromones, tigers, chromatography, multidimensional, gas chromatography, mass spectrometry, olfactometry

## Abstract

In conjoining the disciplines of “ethology” and “chemistry” the field of “Ethochemistry” has been instituted. Ethochemistry is an effective tool in conservation efforts of endangered species and the understanding of behavioral patterns across all species. Chemical constituents of scent-markings have an important, yet poorly understood function in territoriality, reproduction, dominance, and impact on evolutionary biology, especially in large mammals. Particular attention has recently been focused on scent-marking analysis of great cats (Kalahari leopards (*Panthera pardus*), puma (*Puma concolor*) snow leopard (*Panthera uncia*), African lions (*Panthera leo*), cheetahs (*Acinonyx jubatus*), and tigers (*Panthera tigris*)) for the purpose of conservation. Sensory analyses of scent-markings could address knowledge gaps in ethochemistry. The objective of this review is to summarize the current state-of-the art of both the chemical and sensory analyses of scent-markings in wild mammals. Specific focus is placed on sampling and sample preparation, chemical analysis, sensory analysis, and simultaneous chemical and sensory analyses. Constituents of exocrine and endocrine secretions have been most commonly studied with chromatography-based analytical separations. Odor analysis of scent-markings provides an insight into the animal's sensory perception. A limited number of articles have been published in the area of sensory characterization of scent marks. Simultaneous chemical and sensory analyses with chromatography-olfactometry hyphenation could potentially aid conservation efforts by linking perceived odor, compounds responsible for odor, and resulting behavior.

## 1. Introduction

### 1.1. Scope of this Review

To understand the ways in which animals interpret chemical messages, sampling, sample preparation, and chemical and sensory analysis must be performed to accurately define the odors and concentrations of chemicals within the signal. This developing field is limited in the scope of information available about chemosensory analysis of wild animal markings. The use of scent- markings as a method for aiding conservation has been reviewed [1], but lacked definition as to how these scent-marks and their chemical constituents were prepared and analytically characterized.

The objectives of this large mammal and great cat scent-marking review are to: (1) classify different sample preparation techniques for their analysis of scent-markings; (2) summarize existing information on the use of advanced analytical methods on these scent-markings; (3) identify different sensory techniques used to characterize odors of these scent-markings; and (4) classify different sample preparation techniques for the analysis of these scent-markings.

This review provides an overall perspective of literature on the subject of chemical and sensory analysis of large wild mammals, particularly great cats (*i.e.*, leopards, snow leopard, lions, cheetahs, and tigers), scent-markings. Development in the area of sampling and analysis of semiochemicals aids in understanding animal behavior that can be used, for example, toward efforts such as conservation of great cats.

### 1.2. Animal Communication

Communication is a process through which animals use their sensory organs to receive information [2], aiding in the delivery of signals between various inter- and intra-species groups. These signals relay a plethora of information, such as alarm warning, reproductive status and mating, territoriality, and resource signaling [3]. Organisms can communicate through olfactory (chemical), auditory, electro, seismic, and visual communication [4]. The most commonly used method of communication; however, in large, wild mammals is chemical signaling, otherwise known as scent-marking.

Urination, scrapes, and species-specific exocrine secretions are frequently used as modes of chemical signaling for intra- and interspecies communication. Presumably, the chemical constituents of the scent marking convey information about the animal leaving the mark (sender) to the receptive animal (receiver) [5].

Scent-markings require accuracy of olfactory detection to send and receive the correct signal. Scent-markings contain a complex mixture of chemical compounds at varying concentrations based on its chemical message [6]. If an animal wishes to deter an interspecific interaction they can alter the chemical concentrations within their markings to deliver a counterfactual message. An example would be chemical mimicry of pheromones. This false cue/message may encourage attraction of prey species to the territory of predators.

### 1.3. Semiochemicals and Pheromones

Chemicals that act between organisms are called semiochemicals [7,8]. In a system of producer-signal-recipient, the signal (semiochemical) is the central component. Semiochemicals are exocrine secretions, produced by one individual and acted upon by another. Mammalian semiochemicals can be single compounds or mixtures of compounds that are quantitatively variable in coding individual identity based on concentration and specific chemical presence [9,10].

In group living species, for example, it is essential that an individual can recognize members of its social group as individuals and distinguish them from non-group members. [11]. Limited research has been allocated to the chemical characterization of mammalian semiochemicals [9,10], although analytical techniques used to identify semiochemicals in a variety of species have recently been reviewed [6,9]. We build on these reviews by increasing coverage of more large mammals, specifically great cats, and by including sensory analyses techniques of scent-markings.

Semiochemicals can be classified as kairomones or pheromones [9,12]. When the producer and recipient are of the same species, semiochemicals known as kairomones are used for communication. Allelochemicals, are specifically used when a producer and recipient belong to different species, mediate interactions that only benefits the receiver communication and are considered intraspecific and the signal is known as a pheromone [8]. Pheromones are released by one individual and are detected by conspecifics. Pheromones relay impactful messages about sex, species specificity, and reproduction to the receiver [13].

Pheromones are extensively used in territory marking by mammals. Although pheromones are often thought of as odorants (volatile organic compounds), they can be odorless (nonvolatile organic compounds) [13]. Often the volatile odorants are deposited as scents in the animal's dung, urine, scalp, hair, feet, skin, chest and/or breast, and/or may be produced by special glands [6,14]. Examples of special activities for scent dispersal include the chin rubbing of rabbits, check rubbing in pronghorn (*Antilocapra americana*), cheek rubbing and interdigital scrapping in domestic cats, interdigital scrapping in white-tailed deer (*Odocoileus virginianus*), and head rubbing in goats [15–18].

Pheromones are classified into two categories: (1) primers, which prolong a shift in the physiology of the recipient and (2) releasers, which trigger a rapid behavioral response [19]. Primer pheromones generate longer-term physiological/endocrine responses [14]. The course of a releaser is through the nervous system and its primary action generally involves the endocrine system, but is also regulated by the excretory system. Releaser pheromones are involved in four general types of communication: (1) alarm; (2) recruitment; (3) reproductive; and (4) recognition [7].

Alarm substances communicate that there is a possibility of danger. Recruitment pheromones are commonly found in social insects. They are generally employed by worker castes of social insects to guide their nest mates to a food source [7]. Reproductive pheromones come in the form of scents that influence reproductive behavior in many species. These chemical signals can act as an attractant, which links sexes together or increases aggression, or as an aphrodisiac to generate exact aspects of precopulatory or copulatory behavior [20,21].

In many vertebrates mother-young recognition is contingent on chemical cues [22]. Territory and recognition scents are difficult to categorize because sometimes it is unknown if it is a territory scent, a scent that acknowledges social status, or a scent that identifies an individual [7]. For a thorough review of the functionality and origin of pheromones in animals refer to references [7,14,23].

### 1.4. Scent-Markings

Scent-marking is described as the most ubiquitous form of chemical signaling in mammals [5]. Chemical ecology, otherwise known as ethochemistry, is the study of these signals and the interactions they mediate [7]. Chemical signals and their resulting behavioral interactions are multifaceted and varied.

Scent-marks are placed on objects in the environment, frequently in the absence of the receiver, and may only be detected later, in the absence of the signaler [5]. Senders are often not present to reinforce their scent signals and are unaware of whether the mark will be detected and by whom. Scent-marks often degrade before they can be detected, as a result of environmental factors such as rain [11].

To counteract degradation, male mammals generally will remark active scent-markings. Compounds in scent-markings that have longevity under environmental conditions tend to have high molecular weights and low vapor pressures. Some examples of compounds that are found ubiquitously in scent-markings are: squalene, cholesterol, and long-chained carboxylic acids. These compounds are primarily in the secretions/excretions of mammals [24].

**Figure 1.**
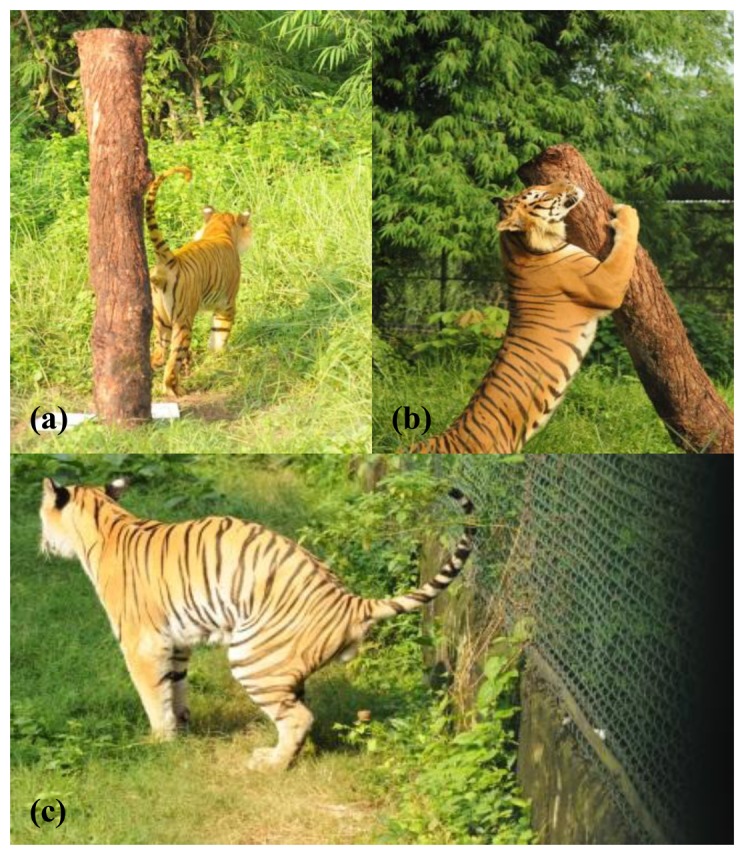
A Bengal tiger (*Panthera tigris tigris*) performing a variety of scent-marking behaviors in its outdoor enclosure at Khayebari Tiger Rehabilitation Project: (**a**) releasing marking fluid; (**b**) clawing/scratching (**c**) defecating.

The most common form of marking is for resource defense territories. Scent-marking by resource holders presents an opportunity for competitor assessment [5]. Scent-marking has long been associated with male intrasexual competition [5,25,26]. Males appear to use scent-marking to obtain territories. Marking frequency is associated with social status and is placed in the areas of the territories where intrusion is the greatest (Figure 1). In some species, males usually leave scent-marks for females, but males often intercept these markings. Females use these scent-markings to assess mate quality through smelling direct body odors [27].

Detection of scent-marks is dependent upon the sensory neurons for olfaction within the vomeronasal organ (VNO) and the main olfactory epithelium (MOE) [13,21]. Universally, mammals detect odorants and pheromones by the nasal olfactory epithelium via the main olfaction system and the vomeronasal organ [13,21]. Sensory neurons that reside in the olfactory epithelium detect a plethora of chemicals. Within the olfactory epithelium there are two types of G protein-coupled receptors (GPCRs): (1) olfactory or odorant receptors (ORs) and (2) trace-amine associated receptors (TAARs) [28]. There are about 800–1500 OR genes that encode GCPRs, which are vital in odorant recognition in the olfactory epithelium [13].

According to the stereochemical theory of olfaction, mammals bind odorants to specific OR sites based on the size and shape of the molecule [29], which results in odor perception [13]. TAARs are a smaller family of receptors that define a specific population of canonical sensory neurons throughout one area of the olfactory epithelium, and are present in a wide variety of vertebrates [28]. It has been suggested that TAARs are located in the nose and have the ability to detect amine pheromones such as isoamylamine, 2-phenylethylamine, and trimethylamine [28]. Thus the olfactory epithelium appears to contain physically separate pheromone receptors than the vomeronasal organ.

The persistence time of the mark is the interval between deposition and the time when the mark can no longer be sensed [11,30]. The persistence of the marks is heavily dependent on two factors: the relatively large size of its molecules and the lipid component [5,11,31,32]. The large molecular mass is thought to result in lower volatility and increased persistence in the environment. The lipid portion of markings is known as a ‘lipid fixative’ [31,32]. In many great cat species it is comprised of free fatty acids, glycerides, esters, and phospholipid [31]. In the absence of this lipid component, aroma substances evaporate expeditiously [33].

### 1.5. Sample Preparation and Chemical Analysis of Scent-Markings

Sample preparation serves an important role in the efficient extraction of components of interest from the sample matrix. The results of this extraction process are later used with analytical instrumentation for target analyte: separation and isolation into constituents, identification, and quantitation [34]. Some biological samples are not suitable for direct analysis and therefore rely heavily on the efficiency of sample preparation and extraction procedures for future analytical analysis [35,36].

Recent advancements in sample preparation and analysis of biological samples can aid in addressing needs and knowledge gaps when applied to scent-markings. Reduced sampling, sample preparation time, and faster, more sensitive and precise analytical procedures have the potential to help scientists working in the field of scent-marking analysis [37].

#### 1.5.1. Sample Preparation Techniques

There are two main approaches to sample preparation techniques; solventless and solvent-based.

##### Solvent-Based Sample Preparation Techniques

Sample preparation methods are categorized by the compound's class, polarity, molecular weight (MW), volatility in which it can be extracted, the physical state (solid, liquid, aerosol and gas), and the analytical instrument used for chemical characterization [35,37,38]. Solvent-based preparation techniques are often used for the identification of peptides and proteins. Peptides and proteins tend to be polar and their MW is typically less than 5 kDa. This allows for techniques such as dried-droplet, double layer, and thin layer techniques to be used in conjunction with matrix-assisted laser desorption/ionization (MALDI) as an analytical method [36,37]. Methanol- and ethanol-based solvents have also been widely used in the sample preparation of lipids in scent-markings [31,39–41]. Solid phase extraction (SPE) has been used for the understanding of pheromone signaling and endocrine communication [42]. Dihydroxybenzoic acid is commonly used in characterizing carbohydrates and polar compounds with a mass greater than 3 kDa [43].

##### Solventless Sample Preparation Techniques

Modern day sample preparation has advanced dramatically in the area of solvent-free extraction processes [34,44–49]. Solventless preparation methods generally require minimum steps, conserve time, minimize the use of toxic compounds, and minimize the interferences and impurities introduced to samples with solvents. In the analysis of biological samples, the most commonly utilized solvent-free techniques are phase preparation methods, which include: solid phase microextraction (SPME), and solid-phase dynamic extraction [35,37,50]. SPME combines sampling and sampling preparation and is useful for non-destructive *in vivo* extractions from biota [51–53]. Reference [37] reviewed advanced methods of solventless preparation.

#### 1.5.2. Analytical Instrumentation

Analytical methods are designed to separate, isolate, identify, and quantify analytes of interest within a sample. There are various techniques and reviews on the separation of these components, specifically in mammals [6,54]. With regard to characterizing scent-marks of wildlife, the most frequently implemented analytical techniques are: gas chromatography (GC) [55], gas chromatography-mass spectrometry (GC-MS) [6,44,56–59], gas chromatography-flame ionization detector (GC-FID) [31,44], GC-time of flight mass spectrometry (GC-TOF-MS), nano-liquid chromatography-mass spectrometry (nano-LC-MS) [40], matrix-assisted laser desorption/ionization- time of flight mass spectrometry (MALDI-TOF MS) [42,60,61], electrospray ionization MS (ESI-MS) [60], gel electrophoresis [62], thin-layer chromatography (TLC) [31,33], gas liquid chromatography (GLC) [31], and tandem MS (ESI-MS/MS) [62].

In GC, the most widely used analytical tool, a mixture of volatile organic compounds (VOCs) is separated into individual VOCs and semi-VOCs, which are eluted out of the GC column at different times [63]. This allows for the quantification and qualification of the compounds within the mixture [63]. Another reason for the common implementation of GC is that it is capable of analyzing volatile compounds that can be detected via the olfactory system. Identifying compounds using GC-MS is more efficient than other detectors because it has an extensive library available with over 200,000 entries (NIST EI-MS database) for comparison matching.

### 1.6. Sensory Analysis of Scent-Markings

Odor detection is a critical constituent in animal interpretation of scent-markings. Inferences into the actual chemicals and odors sensed by animals have been sought through the use of chemical and sensory analytical instrumentation and the use of animals. Rodents have been commonly used to measure the efficacy of the longevity of scent-marks [64–66]. Conservation studies have introduced the use of scent-matching dogs in order to estimate wildlife populations [67–70]. The use of simultaneous chemical and sensory analyses is an area of limited study with regard to mammal scents.

In recent years, the introduction of application-specific sensor array systems, otherwise known as “electronic noses”, were developed and combined with GC, MS, and infrared spectroscopy to mimic the sensitivity of the human (*Homo sapiens*) olfactory system's measurement of volatiles [71]. This can be applied to broaden the understanding of how animals use olfactory cues to understand chemical messages.

#### 1.6.1. Animal Detectors

Over the last several decades, scent-marking odor classification of mammals has been limited in its ability to fully characterize the odorous volatile organic compounds (VOCs) within the marking and to detect their presence in the wild. Often this identification is performed via conspecific confirmation. Mice have been the primary models of olfactory detection and interpretation of markings, such as in deciphering the age and reproductive messages in urine [27,64,72,73]. Mice have also aided in the identification of 2-phenylethylamine as one of the kairomones responsible for avoidance behavior.

Dogs have also been used in the estimation of wild animal populations based on individual scent-mark recognition [68,74]. The use of animal detectors, however, instead of sensory instrumentation can limit the amount of information acquired from the marking.

The human nose has been an olfactory detection system in various studies of animal pheromones. When m-cresol, 2-heptylpyridine, hexanal, (Z)-6-dodecen-4-olide, and α-terpineol were present in high concentrations, they were identified by human nasal detection as the compounds responsible for the pleasant herbal smell of bontebok (*Damilscus dorcas dorcas*) interdigital gland secretions [75]. The sensitivity of the human olfactory system permitted the detection of reproductive semiochemicals, 5α-androst-16-en-3-one (H5-down), 505β-androst-16-en-3-one (H5-up), and 3α-androstenol in pigs (*Sus scrofa*) [9,76]. Human sensitivity toward these compounds has been used to develop theory that such compounds could also be human pheromones [76]. Studying kin recognition olfactory cues in human neonates has determined that pheromones from their mother's breasts and underarm pad are used to distinguish their mothers from other women [77].

Simple human nasal detection was performed for the determination of the characteristic odor of tiger marking fluid [30,33]. They described the odor as that of basmati rice caused by 2-acetyl-1-pyrroline (2-AP). This conclusion was based on personal and cultural experiences with this food item. This type of identification is useful, yet it could limit identification of all potential odorous compounds that may be contributing to the characteristic odor in highly complex scent mixtures.

#### 1.6.2. Simultaneous Sensory and Chemical Analysis

The implementation of simultaneous chemical and sensory analyses is the modern approach to investigating the odors, tastes, and visual appearance of chemical compounds in biological samples. Based on their detection mechanisms, these systems can be classified into several categories, including chemical sensors, biosensors, GC-based systems, MS-based detectors, and hybrid GC/chemical sensors. Specifically, ‘electronic noses’ (‘e-noses’), multidimensional gas chromatography-mass spectrometry-olfactometry (md-gc-ms-o), ‘electronic tongues’, and visual analyzers are a few types of biosensory technologies available for the characterization of biological compounds. The reaction between odor molecules and the target sensing materials on the sensor surface triggers changes in mass, volume, or other physical properties. This reaction is then converted to an electronic signal by a transducer.

Widely used types of transducers include optical, electrochemical, heat-sensitive, and mass-sensitive. Some common chemical sensors are: surface acoustic wave sensor, quartz crystal microbalance sensor, metal oxide semiconductor sensor, and polymer composite-based sensor. An ‘e-nose’ is an instrument that is designed to mimic the function of the natural nose. By definition, it uses a sensor array to not only detect but also discriminate among complex odors [71,78,79].

The ideal example for the detection of odors is the mammalian nose because of its ability to evaluate with both high sensitivity and specificity. Olfactory receptors make these properties possible, as they support combinatorial detection of odors at trace levels (e.g., 10^−7^ to 10^−11^ M in humans) [80,81]. Exhaustive efforts have been devoted to exploiting these receptors in association with some electronic devices to develop biosensors that truly mimic biological noses [82–85].

The detection mechanism of these biosensors is based on the specific interaction between olfactory receptors and odorant molecules. Biosensors have been known to demonstrate better detection selectivity than chemical sensors. The ‘bio-sniffer’ is another example of a type of biosensor developed for VOC detection that is based on biochemical reactions between a biomolecule and a VOC, or a chemical reaction catalyzed by biomolecules [86,87].

md-gc-ms-o is capable of removing the interference effect from non-target components. This system allows the users to separate components of interest, identify character defining compounds, and identify those components using modern mass spectral techniques [51,88–94]. MD-GC-MS-O allows for the simultaneous analysis of compounds with the human nose as an odor detector and the MS as the chemical analyzer [93,94]. Specifically, the MD-GC-MS-O is used in the identification and characterization of VOCs and semi-VOCs in a variety of biological systems.

A few examples of research that have been performed using MD-GC-MS-O and simultaneous chemical and odor identification are: identification of compounds responsible for the characteristic odor of livestock and poultry manure and rumen of beef cattle; association of a specific odor with a volatile compound; the role of particulate matter as a carrier of odor; characterization of kairomones and characteristic odorants released by insects; and quantification of nutraceuticals in wine [51,89–98].

This analytical tool is a state-of-the-art technology that is particularly suited for identification of chemical-odor association. This instrument can be used to explain the association between VOCs and their odors in wild mammal secretions and excretions. MD-GC-MS-O is capable of determining the concentrations of these compounds and evaluating the intensity and aroma of the odors of the entire scent-mark. Identification of compounds responsible for specific odors and signaling could aid wild mammal conservation, and it would serve in giving some insight into how and why animals are detecting these scents.

## 2. Methodology of the Literature Review

Articles were obtained through searches on Science Direct, Academic Search Premier (EBSCO), and Google Scholar article databases. Keywords and phrases that were used in the searches included: “conservation”, “GC-MS”, “GC-MS-O”, “gas chromatography”, “chromatography”, “endangered species”, “odor”, “chemosensory”, “simultaneous chemical and odor analysis”, “panthera”, “elephas”, “odocoileus”, “TAARs”, “olfactory receptors”, “scent-marks”, “urine”, “feces”, “mammals”, “scent-marking”, “conservation”, “animals”, “volatile organic compounds”, “sample preparation”, “analytical techniques”, “large mammals”, “pheromones”, and “marking fluid.” Articles selected for this review focused on the use of modern analytical techniques to identify and/or quantify chemical compounds detected in scent-markings of large wild mammals and great cats for the purpose of sensory and chemical identification, conservation, behavioral understanding, and evaluation of sampling and sample preparation effectiveness.

Citations from the initial search were downloaded into EndNote, a reference management database. Duplicate citations were removed. Assessment of the identified studies for relevance was based on a standardized criterion developed by all co-authors: (1) the focal animal reported was a large wild mammal; (2) analytical techniques were utilized for chemical identification of scent marks; (3) sample preparation was defined; (4) the articles were peer-reviewed; (5) if sensory analysis was performed the method needed to be clearly defined; and (6) the co-authors had no objections, such as quality or topic focus of the articles.

If any of the five criteria were not met, the reference was omitted. For articles that remained in the review after applicability and quality selection, data were summarized and reported. Data extraction from these articles was completed by one reviewer and when uncertain this reviewer consulted with the other authors. Data extracted from the research articles included: (1) sample preparation technique; (2) analytical methods; (3) animal species; (4) sensory analysis approach; (5) relationship to conservation; and (6) scent-markings being collected. Conclusions were based on a summary of the data.

## 3. Results and Discussion

### 3.1. Chemical and Sensory Characterization of Scent-Markings in Wild Mammals

#### 3.1.1. Sampling and Sample Preparation

This section summarizes sampling and sample preparation methods performed for the analysis of scent-markings of large mammals. It discusses solvent-free and solvent-based extraction methods and the advantages and disadvantages of these methods. The sampling and sample preparation section also explains the similarities and differences between the uses of various techniques for the identification of chemical constituents in scent-markings.

##### Solvent-free Extraction

Solvent-free extraction methods often reduce sample preparation time and eliminate multiple step procedures for the extraction of a component from a sample. Conventional solvent-free extraction methods implemented for wild mammal scent-marking characterization included: headspace extraction, direct injection, precolumn heaters, solid phase extraction (SPE), stir bar absorptive extraction (SBSE), and solid phase microextraction (SPME). Headspace extraction is the process of transferring a substance from a solid or liquid matrix to the vapor phase by heating, and removing analytes from the headspace in a carrier gas [99]. Direct injection is the direct insertion of an aqueous solution or aqueous extract from a sample matrix onto a GC column [100]. The precolumn heater (PH) technique is a solvent-free method to collect volatile compounds. It consists of a glass cylinder heated to 100 °C with N_2_ being released simultaneously and driving the volatile material into a needle at the end of the cylinder [101,102]. SPE is performed by adding the test solution or solvents through a sorbent which is packed in a column and separation of both phases then occurs [103]. SPDE has been used to identify sulphur-containing hermiterpenoids responsible for the unique odor of maned wolves (*Chrysocyn brachurus*), when SPME was ineffective [104]. SPME is a combined sampling and sample preparation method that utilizes a fused-silica fiber coated with a thin polymeric film to passively diffuse compounds in a sample onto the SPME fiber via adsorption, absorption or capillary condensation [52]. In some cases, SPME extracts and collects samples from various environments without additional preparation before analytical separation [52,92].

Headspace extraction results in the emissions of volatile compounds to the headspace, and thus provides some information about the fate of semiochemicals based on their physicochemical properties. This is particularly important when providing evidence of an animal's ability to identify compounds in the air from extreme distances. These volatile compounds are essential to our comprehension of animal communication. Headspace autosampling extraction of gases emitted from urine can provide information on compounds potentially detected by passing animals, specifically lions [59]. Headspace extraction can reduce sample preparation time and reduce impurities associated with solid or liquid matrix of a sample [49]. Reference [105] performed adequate headspace extraction on Asian elephant (*Elephas maximus)* blood volatiles in 35 min in comparison to other lengthier procedures.

VOCs in sternal secretions from koalas (*Phascolarctos cinereus*) were analyzed using a solvent-free technique [106]. The sternal secretions were collected and pipetted onto filter paper without solvents or additional extraction techniques. This extraction method was inexpensive, rapid, and helped to find three additional nitriles (isobutyronitrile, 2-methyl-, and 3-methylbutyronitrile) suggested to be involved in odor cues, but never before detected [106].

The PH technique allowed for the identification of compounds in the interdigital glands of reindeer (*Rangifer tarandus)* [101,102] and was used to identify a recognition scent in the tarsal glands of male black-tailed deer (*Odocoileus hemionus columbianus*) and reindeer. This scent is recognized through tugging and licking the tarsal gland and is used to identify individuals by the scent associated with them [107]. The chemical responsible for the scent is cis-4-hydroxydodec-6-enoic acid lactone.

Solid phase dynamic extraction (SPDE) is an extraction process that can be utilized at ambient room temperature to extract semi-VOCs. When coupled with an automated sampling system that can regulate temperature, a higher number of volatile compounds can be extracted. Using a SPDE needle internally coated with a modified activated charcoal-polydimethylsiloxane (AC-PDMS) allowed for a small sample size of 0.5 mL of *Strepsirrhini* urine for characterization. This urine characterization led to the phylogenetic construction of the *Strepsirrhini* suborder [45]. Utilizing SPDE reduced the extraction time in comparison to a solvent-based procedure [45].

Stir bar absorptive extraction (SBSE) techniques have been advantageous in measuring small sample sizes and diluted media [108]. Volatile and semivolatile substances from aqueous and gaseous media have been extracted using a polymer-coated magnetic bar (Twister ™) [108–110].

The polydimethylsiloxane (PDMS) coating on the stir bar and constant stirring agitation allows for a more precise and reliable extraction, and decent analytical precision [108]. In SBSE, generally the phase volume is between 24 and 100 μl, exceeding the solid phase microextraction technique which is typically 0.5 μl. A few studies have utilized SBSE in the detection of 26 volatile compounds of preputial glands of rodents [108,111]. Nonanol, benzaladehyde, several ketones, pyrazines, sulfur compounds, and heptanones have been reported as volatile characteristic compounds in mammal species using SBSE [108,111].

**Figure 2.**
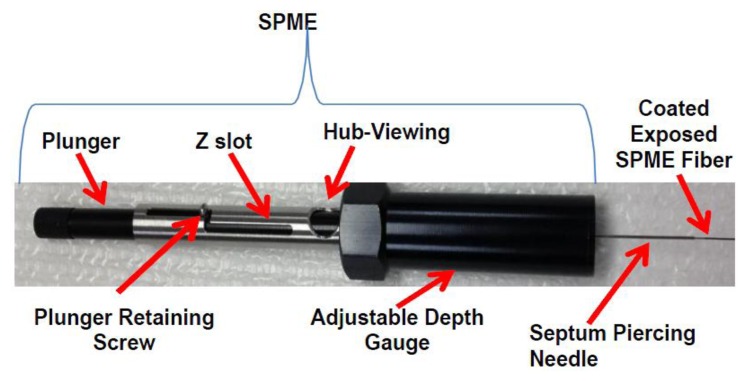
A manual SPME holder. SPME can be also used with any mainline autosampler for automated sample preparation.

**Figure 3.**
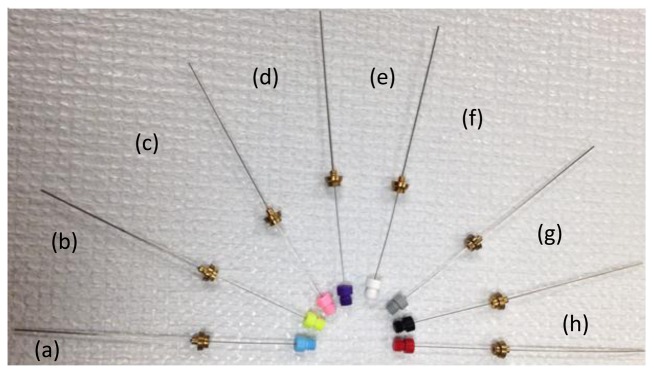
A variety of solid-phase microextraction fibers with different coatings used for the identification of non-polar and polar compounds, volatile odorous compounds, and/or compounds of different molecular weights: (**a**) 85 μm PDMS (**b**) 70 μm Carbowax/divinylbenzene (CW/DVB) (**c**) 65 μm PDMS/DVB (**d**) 50 μm CW/templated resin (**e**) 85 μm polyacrylic (**f**) 50/30 μm DVB/Carboxen/PDMS (**g**) 75 μm Carboxen/PDMS (**h**) 100 μm PDMS.

Solid phase microextraction (SPME) is particularly suited for characterization of volatiles from biota. SPME can be used for *in vivo* extractions of volatiles. SPME is a solventless extraction technology that incorporates fibers of assorted coatings and a fiber holder (Figures 2 and 3) that is either directly (e.g., by submersion in liquid) or indirectly (e.g., headspace) exposed to a sample. Different fiber coatings (Figure 3) can be used to optimize the type of compounds to be extracted from the sample. Volatiles and semi-VOCs passively diffuse onto the SPME fiber via adsorption, absorption or capillary condensation. SPME fiber coatings have very high affinity for VOCs and semi-VOCs [53].

Thus, the sampling results in high preconcentration and enrichment of compounds that did not require use of solvents and additional steps. Specific SPME coatings can be used for optimization of extraction processes favoring certain groups of compounds varying by MW, polarity, and functional groups. Often fibers with Carboxen polydimethylsiloxane (Car-PDMS) coating are used for the detection of VOCs with low MW. Divinylbenzene/Carboxen/PDMS coating is used on a broad range of analytes, specifically volatile and/or semi-volatile compounds. SPME combines sampling and sample preparation to minimize the sample preparation step with a process that is simple, reusable and efficient.

There are relatively few publications that report the use of SPME for characterization of scent- markings of large wild mammals [44,90], However, SPME has its strengths and challenges in regard to sampling, sampling preparation, and analysis of biological samples. SPME has been found to be effective in the analysis of trace levels of analytes in the urine of Strepsirrhine families [112].

Automating headspace extraction with SPME was useful and a non-invasive method for monitoring reproductive status via the urine in elephants and other species [105]. African elephant (*Loxodonta africana)* urine analyzed with SPME used a chiral column to detect the pheromone, frontalin [44]. When SPDE and GC-MS analysis was performed with headspace extraction, however, it made the number of steps in the sample preparation and analysis of maned wolf urine diminutive in comparison to solvent-based techniques [104].

The use of ultrasound as a tool for compound separation has proven to be less effective than SPME. In the case of giant panda (*Ailuropoda melanoleuca*), ultrasound was used for 15 min to separate anogenital gland secretions from tampons [113]. The extract was then left to settle for 5 h resulting in 5 less VOCs in anogenital gland secretions than previous studies using SPME [113,114]. In the analysis of tiger urine and marking fluid, the use of headspace sampling with a ‘sample enrichment probe’ containing a 28 mg PDMS rubber, reduced solvent preparation time and was possibly two orders of magnitude more efficient than SPME in general practice, dependent upon application [47,115]. The volume of the coating of an extraction fiber whether SPME or sample enrichment probe (SEP) determines the level of sensitivity and rate of extraction from a sample matrix [34]. In comparison to SPME the volume of the coating and extraction surface area of an SEP PDMS rubber is larger, potentially resulting in superior extraction efficiency.

##### Solvent-based Extraction

Territory and recognition scents are difficult to categorize because the scent may indicate territorial boundaries, social status, or individual animals, or incorporating all three factors [7]. Social status information is often associated with urination. To date, the majority of mammal urine extractions are accomplished via solvent-based extractions. Solvent-based extractions generally require a series of procedures and are time consuming. Multiple bioassays and fractionation processes made the methods for detection of cycle stage, parturition, and estrous of elephants an extensive procedure [116].

Methanol extraction of koala sternal gland secretions required upwards of 8 hours [117]. The extraction process for black buck (*Antelope cervicapra)* urine used dichloromethane as the solvent and liquid N_2_ to condense the extracted sample. This resulted in a total sample preparation time that was less than 1 h [118]. Solvent-based methods may have an impact on the chemical composition of a sample due to the interactions of chemicals within the scent mark and the solvent (or solvent impurities) used to extract the compounds of interest. The addition of methanol after sample collection and chloroform during tiger urine sample preparation, may have altered the results [31].

**Figure 4.**
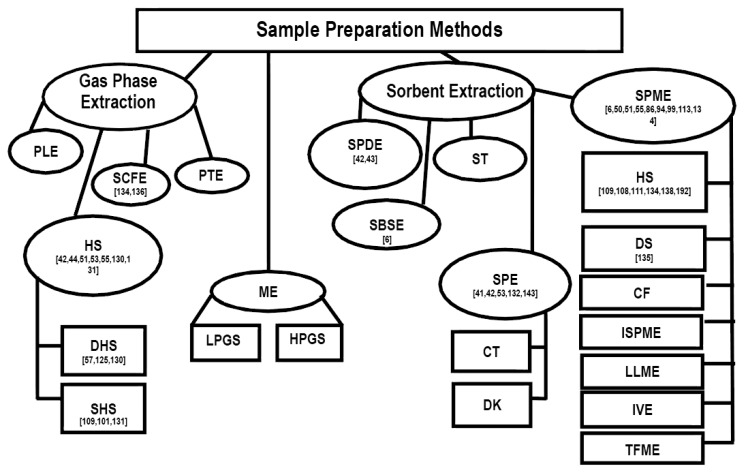
Summary of sampling preparation techniques with references used for chemical and sensory characterization of scent-markings in wild animals.

Summary of sampling and sample preparation techniques with references used for the chemical and sensory characterization of scent-markings in wild mammals is presented in (Figure 4). To date, the most frequently used sampling and sample preparation methods are: (1) solid-phase microextraction/headspace extraction; (2) solid-phase dynamic extraction; (3) static headspace extraction; and (4) solid-phase extraction.

It appears that in the last decade there has been a rise in the implementation of SPME for the sample preparation and sampling of scent-marks (Figure 4). This increase in SPME use may be due to the fact that it does not require the use of a solvent, can reduce sampling and sample preparation time by combining the two procedures, is very transportable for field analysis, and is highly efficient in extracting compounds of interest from biological samples [119].

### 3.2. Chemical Analysis

Research in chemical signaling plays an important role in the conservation of many endangered large animals. This section summarizes analytical methods performed for the analysis of scent- markings of large mammals. The use of various GC- and high performance liquid chromatography (HPLC)-based techniques with an assortment of detectors is summarized with the advantages and disadvantages of each method.

#### 3.2.1. Gas Chromatography

Gas chromatography (GC) is a very useful analytical technique for the analysis of mammal scent- markings (Table 1). The use of GC resulted in finding high proportions of steroids and other chemicals that were not previously reported in gray wolf (*Canis lupus)* urine and feces volatiles [120]. Another example of the good utility of GC was reported in its use to characterize VOCs in human biological secretions and excretions. GC was fairly good at reproducibility in analyzing human urine, breath, and blood [46].

GC combined with a detector allows for the identification of compounds within the sample. The most commonly used detectors were: MS, FID, and FT-IR. MS was the most widely used because of its capability to perform a spectral search and match for over 200,000 compounds within its spectral library. Also, MS detection was preferred with GC analysis because of its compound identification abilities and sensitivity [121,122]. The GC-MS spectral library comparison made chemical identification of Strepsirrhine families' urine uncomplicated [45,123].

While GC-MS is a well-established and often preferred technology for detecting volatile compounds with MW below 300, it is not ideal for the detection of higher MW compounds [113,118]. The use of GC-MS resulted in the detection of low MW and nonvolatile compounds of giant panda (*Ailuropoda melanoleuca*) anogenital gland secretions, urine, feces, and blood serum [113]; all of which were not readily detected by HPLC [127].

In the case of urine from gray wolves, notable peaks from the GC were identified through matching GC retention times and MS spectral patterns [133]. The use of GC-MS for the extraction of aromatic compounds in urine and feces of *gray wolves* was deemed efficient [132]. SPME-GC-MS combined with GC-Pulsed Flame Photometric Detector dichloromethane extracts coupled with GC-FID resulted in the identification of 103 compounds in urine, feces, and anal gland secretions of African wild dogs *(Lycaon pictus)*.

Out of all of the 11 species-specific compounds, 8 were confirmed. The confirmed compounds were: 1,3-propandiol, N,N-dimethylacetamide, 1-methyl-2,4-imidazolidinedione, 1-methylimidazole-5-carbox-aldehyde, and quinazoline. The aforementioned compounds were at three times the level in urine than feces [124]. This analytical method, although beneficial, was lacking in its ability to conclude chirality issues with identified compounds and the position of double bonds in unsaturated acids.

Although GC is the modern system for separations and chemical composition determination, the use of variable detectors, in conjunction with the GC, may impact the ability to quantify or qualitatively define scent-markings. While GC-MS analysis allowed for quantification of the compounds in the scent-markings of brown-mantled tamarin *(Saguinus fusciollis)*, compounds with concentration levels of 0.01% were omitted from analysis, possibly excluding the incorporation of specific pheromone or semiochemicals that are essential in animal communication but present in very low abundance [135]. The use of GC-MS [118] resulted in detecting volatile compounds in black buck urine that had a MW of less than 300. White-tailed deer urinary lactone, (Z)-6-dodecen-4-olide, previously found in the tarsal gland of deer were not detected via GC-MS [128].

In addition, nondistillable compounds in the tarsal gland were also not identified through GC-MS detection [18]. In the case of bobcats (*Lynx rufus)*, MS and retention time identification allowed for first time confirmation of compounds in urine [143]. Nevertheless, the combination of the two methods of detection provided a true confirmation and multiple assessments of urinous compounds.

GC-based analyses had some additional drawbacks such as sample dehydration/alteration. Dehydration was observed when characterizing koala sternal gland secretions [106], *i.e.*, dehydration of the oximes occurred during the desorption of the swab in the GC injection port. In the identification of castoreum composition in the American beaver (*Castor canadensis*), GC analysis may have impacted the analysis of highly volatile phenol constituents [140]. Previous studies used alcohol and additional ‘basic materials’ with fractionation for extraction and alumina chromatography for analysis. Using this method, cis-Cyclohexane-1,2-Diol was identified in beaver castor sacs [145]. GC-FID is highly efficient in the quantification of chemical compounds. GC-FID in combination with GC-MS has been efficient in the identification of 103 compounds in African wild dogs. It has been suggested, however, that nonvolatile compounds in urine of Strepsirrhine families may not be detected via GC-FID [131]. The interdigital and tarsal scent compounds of black-tailed deer were identified through retention time and not with a mass spectral library database because gas liquid chromatography-flame ionization detector (GLC-FID) and GC were employed [107,125,126].

Elephants have been a major focal animal in the area of scent-marking and its role in reproduction and socialization. They have been used to understand how scent-marking impacts mating and interaction of males and females of various ages and social levels within herds [136,142,146,147]. Male and female African elephants have developmental differences in chemosensory signal processing [148]. The exhibition of musth pheromone (frontalin) released by male elephants has been known to elicit female sexual responses to the male [136]. The use of SPDE and SPME in conjunction with chiral column GC-FID and GC-MS were useful in the detection of frontalin [44]. Ketones such as 2-butanone, acetone, 2-pentanone, and 2-nonanone have been quantified using GC-MS and showed elevated levels during all periods of musth [142]. A series of alkan-2-ones and alkan-2-ols were identified in the urine of African elephants using GC-MS [146]. It was suggested that after performing analysis that GC-MS could serve as ‘time-release chemical signals’ to conspecifics [36,149].

For several chemical component identifications, a combination of capillary GC with Fourier-transformed infrared spectroscopy FTIR was essential for accurate identification of *gray wolf*' urine and feces volatiles [133]. MALDI has been used for the confirmation of the precursor pheromone felinine in the urine of domestic cats [61].

### 3.3. Sensory Analysis

A three step process is needed to fully comprehend the role of cues in scent-markings in animal behavior. First, an understanding of which chemical constituents constitute the marking must be determined. Next, an odor characterization of these specific compounds must be performed. Lastly, a behavioral analysis of how the animal reacts to these specific odorous compounds to determine the relationship between behavior and scent must be completed. Without the input of sensory analysis, the interpretation of cues in scent-markings can be limited. The use of the human nose for sensory analyses, as opposed to the use of animal olfactory sensing further complicates this process. This section summarizes the limited information available on the use of chemical and sensory analysis for the characterization of large mammal scent-markings (Table 2).

#### 3.3.1. Electronic/chemical

GC-MS were able to generalize all compounds in spotted hyena *(Crocuta crocuta)* as being responsible for eliciting behavioral responses without detecting specific odorous compounds [141]. This study measured concentrations of VOCs from animals believed to be of different social status and age without the use of olfactometry. These results limit the amount of information associated with the odors that are being detected by the animal.

An ‘electronic-nose’ (E-nose) indicated that VOCs emitted from the body vary with age, diet, sex, physiological status and genetics (Table 2). The main findings in reference [151] are that electro-olfactograms and E-noses can act with the same specificity as the human nose in the detection of volatile compounds and may be applicable in environmental studies.

#### 3.3.2. Animal Detection

Animals are frequently the objects of sensory evaluation (Table 2). Gray wolves return to their territory boundaries every three weeks to re-mark with various scent-markings, which are below detection level after 23 days, to counter the effects of the environment [152]. The detection of these markings is dependent upon how long the compounds in the marking remain odorous. The use of conspecifics, however, to detect olfactory changes in the scent marks of other brown-mantled tamarin made it impossible to qualitatively measure changes [135].

Odor detection thresholds for humans are different for each chemical (i.e., high concentration of virtually odorless compounds does not elicit any response). The same principle is thought to apply in wild mammals. In complex mixtures of scent-markings reside distinct odorous compounds responsible for the longevity of its scent availability. An example of a compound that constitutes a large mammal scent-marking is cyclohexanone. Cyclohexanone elicits flehmen responses from sub-dominant females, but in males there is no response [105]. Elephant detection of cyclohexanone in musth has led scientists to suspect that some musth signal messages in elephants may be single compounds [105]. In the case of cyclohexanone, with a boiling point of 161 °C and a slow volatilization period of hours is responsible for a relatively longer lasting signal than compounds of lower MWs.

Persistence of scent-markings in the environment has been recorded at a wide variety of lengths. In the case of dominant male mice, urine has been avoided by other males for up to 72 h. Klipspringer antelope (*Oreotragus oreotragus*) have scent marks that remain active for as long as 7 days [153]. Scent marks disappear in dwarf mongooses (*Helogale parvula)* after 10 days and in hamsters (*Mesocricetus auratus),* for 100 days. Even humans, however, can detect scent from anal gland marks of hyenids after 1 to 6 months [5]. Humans have utilized nasal detection to survey snow leopard (*Panthera uncia)* territories and marking behaviors by differentiating the age of different urine and scat markings over a period of months. Frequency of marking coincided with the winter/early spring mating season. This marking rate potentially serves to maintain awareness of conspecific presence and also distance between snow leopards [154].

### 3.4. Simultaneous Chemical and Sensory Analysis

#### 3.4.1. Multi-dimensional-Gas Chromatography

Multi-dimensional-gas chromatography (MDGC) has previously been defined as, “the process of selecting a (limited) region or zone of eluted compounds from the end of one GC column, subjecting the zone to a further GC displacement” [121]. Two-dimensional chromatography utilizes two independent GC ovens equipped with proper switching system and column setup. Separation in multi column chromatography occurs by using (a) two columns with different polarity which are connected in series where the whole sample is eluting from the first to the second column; (b) two columns with different polarity connected in series that satisfy the conditions of orthogonality (*GC*×*GC*) (in this instance the whole sample is eluted from the first column to the second column in some specific time frame); and (c) by using practices, where only a small part of the sample elutes to the second column either via backflash, foreflash, and heart-cut [155]. Backflash is a method, where the specific portions of the sample eluted from the second column were previously washed from the first column by switching the direction of carrier gas flow to the opposite direction [155]. Foreflash is used for the removal of remaining solvent, derivatization agent, or other additives [155]. Heart-cut allows the assignment of one or more fractions from the first dimension to the second dimension with a different polarity. Transferring of the sample to the second dimension is carried out by an on-line cutting, which allows transfer for only specific analytes [156].

A series of detectors can be used for two-dimensional GC: flame ionization detector (FID), electron capture detector (ECD), atomic emission detector (AED), nitrogen-phosphorus detector (NPD), and olfactory detector and mass spectrometer (MS) [157,158]. MDGC can be combined with olfactory analysis in the form of an MD-GC-MS-O for the purpose of simultaneous sensory and chemical analysis.

The characteristic or overall aroma of a sample is an intricate combination of various odorants. Simultaneous analyses can potentially identify links between certain scents and the exact chemical compounds causing them. Simultaneous chemical and sensory analyses have the potential of linking both chemical and sensory analyses that are often analyzed independently. MD-GC-MS-O can be described as a two-way split detection system. In this arrangement, compounds are quantitatively trapped in a capillary column loop, which isolates them online from preceding and following peaks, and splits the target region into the second column for effective resolution from interfering matrix compounds [159]; this allows for MS and/or olfactory analysis. A small split flow (∼10%) to the MS detector achieves correct timing to ensure target trapping in the loop which must be sufficiently cool to retain the trapped compounds of the target region [160]. Multidimensional GC-MS was applied to sensory and chemical characterization of odorous gases of swine manure and isolation of *trans*-resveratrol in red wine [89–91,96].

**Figure 5.**
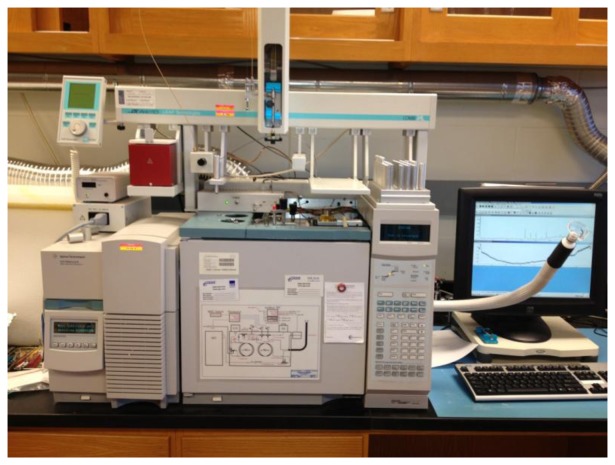
Multi-dimensional gas chromatography-olfactometry system at Iowa State University.

Simultaneous chemical and sensory analysis is very rarely performed in the area of wild large mammal scent-markings. The only instances of sensory analysis were the use of conspecifics after chemical identification [5,42,55,131,161]. GC-MS-O was used to identify characteristic odorous compounds that were in low abundance in a complex mixture of VOCs from various biological samples (urine, breath, feces, and sweat) in humans [135]. Early development of human breath sampling and analysis protocol for clinical settings began through the practice of GC-MS-O instrumentation [138]. GC-MS-O (Figure 5) has also been used to determine odorous compounds released by humans suffering from various illness, such as cancer [138].

It has been reported that olfactory receptors in biosensors are more sensitive detectors of ligands than GC-MS and chemical “noses” [151]. An E-nose is considered a real-time detection technology. This also means that it can be used side-by-side with another system such as a GC-MS. E-noses, however, lack biorecognition stability and portability.

Electro-olfactograms (EOG) are “electrical potentials of the olfactory epithelium that occur in response to olfactory stimulation” [162]. ‘EOGs are the sum of generator potentials of olfactory receptor neurons’ [162]. An electro-olfactogram does not provide information about, or molecular basis of, olfaction without molecular analysis. Another type of biosensor, luminescence optical assay, lacks the ability to detect compounds that do not have low detection limits. This limits the range of compounds it is capable of detecting.

### 3.5. Chemical and Sensory Characterization of Scent Markings in Great Cats

Great cat markings have been studied to aid in conservation, specifically focusing on territoriality, dominance, and reproduction (Table 3) [31,33,41,59,130,163–165]. Great cats use scent-markings as a method for distinguishing amongst other conspecifics and neighbors, as territorial boundary markings, and as reproductive condition indicators. Although there is limited information about the analysis of great cat scent marks, conclusions can be deduced and used to aid in conservation.

#### 3.5.1. Characterization of Great Cat Scent-Markings

Behavioral studies of free-ranging tigers have determined that marking functions to establish and maintain territorial boundaries and advertise female reproductive status [166] (Table 3). There has never been a study, however, that analyzed changes in scent-mark composition over the reproductive cycle of tigers. This would help to identify why these markings are presented with such frequency during proestrus. The main function of cats' sense of smell is to decipher their own scent marks from those of conspecifics, stimulate exploration, and to defend territories [195].

The focus of previous studies has been on identifying total compound composition, neglecting the study of olfaction's relationship to scent-mark identification by animals. Application of MD-GC-MS-O has the potential to measure the influence of odor in scent-marking detection in species that use chemical cues as their communication method.

Scent-mark constituents and/or behaviors have been analyzed in snow leopards, puma*,* African cheetahs, Indian leopards (*Panthera pardus fusca*), and African lions (Table 3). Pumas, leopards, and cheetahs do not contain a lipid component in their marking fluid, unlike in tigers and lions [127]. 2-acetylfuran, acetaldehyde diethyl acetal, ethyl acetate, dimethyl sulfone, formanilide, urea, and elemental sulfur were identified in cheetah urine [6,196]. It has been suggested that elemental sulfur may be a cheetah pheromone, however further research is required [6]. Scent-marking behavior and markings (feces) in snow leopards, pumas, cheetahs, lions, caracals, tigers, mountain lion, and lynx was used to determine taxonomic separation and phylogenetic classification between cat species [174,197]. Common procedures used to chemically characterize scent-markings include headspace extraction and solid-phase microextraction for sample preparation and GC, GC-MS, LC, and TLC for sample analyses [41,198,199]. Previous research suggests that the polarity of a solvent, specifically nonpolar solvents, as well as the geometric isomerism of a semiochemical molecule influences elution order of semiochemicals using gas liquid chromatography [200]. This work specifically focused on alkene elution. The elution orders of simple alkenes, especially those removed from the chain termini, eluted later than the cis-alkenes when the solvent was nonpolar. This has aided in understanding the configuration of total ion chromatograms (TIC). Within the past decade, GC-MS has been the leading technology for scent-marking characterization in great cats.

Chemical composition of semiochemicals of Bengal tigers, African cheetahs, and pumas have been analyzed [33,41,47,68,69,161,166,183,201]. Tiger marking fluid (MF), urine, and feces are the known sources of chemical communication in tigers. Analytical methods implemented in the detection of tiger semiochemicals include: GC, TLC, and GC-MS. Ninety-eight volatile compounds have been identified in the MF of Bengal tigers [47]. It has been assumed that tigers use these volatile and non-volatile markings to convey olfactory signaling. What is inhaled, however, and how it is processed has not been completely identified [33,47,167]. 2-acetyl-1-pyrroline has been the only compound associated with the characteristic odor of tiger marking fluid [33]. The identification of this compound in Bengal tigers has been achieved by aroma identification; however the lack of a ‘sniff GLC’ or GC-MS-O has prevented its analytical confirmation [33,47,167]. Burger et al. were never able to confirm 2-AP in Bengal tiger MF or urine [47]. The methods for the identification of 2-AP aroma was based on the addition of hydrochloric acid for acidifying and preventing volatilization, followed by the addition of alkali for aroma identification, and addition of 2% KI to cleave the reactive methyl ketone group of the 2-AP molecule [33,202]. These steps were followed by odor identification based on human olfaction, but its presence has never been confirmed with analytical tools. References [203,204] suggested that the use of human simple olfactometry detection produces limitations making “it very difficult to appreciate the sensory ranges of animals.” Though 2-AP is a characteristic odor compound of Bengal tigers it may not be the only compound associated with the overall characteristic odor [205].

The use of GC and LC has enabled characterization of MF from Bengal tigers, specifically its lipid component, VOCs, and a general characterization of MF odor, similar to that of basmati rice. The use of MD-GC-MS-O could potentially define all odorous compounds and provide an all-encompassing and accurate overview of odorous compounds responsible for eliciting behaviors and tiger identity.

In the case of the Bengal tigers, two methods have identified the total lipid and urinary portions of the MF, *i.e.*, TLC and GC-MS. TLC has been used for quantitatively determining lipid composition of Bengal tiger marking fluid [31,129], and GC-MS has been utilized to quantify both lipid and urinary components of Bengal tiger MF [47]. Comparison of differences in the chemical composition and concentrations of marking fluid and urine of subspecies of tigers have never been conducted.

The sebaceous glands contribute to the production of lipocalin protein molecules and fixative lipids in tigers which aids in the long term persistence of marking fluid (MF) in the wild [31]. Bengal tiger marking fluid compounds have been primarily identified using GC column retention time [31]. Retention times are not ideal as chemical co-elution can occur particularly in complex scent-related matrix. The age of the sample and presumed loss of compounds over time can make it impossible to detect volatile compounds, specifically 2-AP using GC-MS [33].

Genetic characterization and definition of Siberian tigers (*Panthera tigris altaica*) and the Amur leopard (*Panthera pardus*) are needed to restore their populations. Previous felid research has led to their species and sex identification from fecal and hair samples [169]. Reference [169] used scent-matching dogs to determine that each tiger has uniquely identifying scent-marks that can be detected by dogs 76% of the time [169]. This indicates that there is a strong association between characteristic odor and chemical composition of scent marks. Feces have also been used as an indicator of tiger population numbers and territorial distribution [68]. Scent-markings have also been used to determine population densities of tigers and pumas.

The volatile constituents of lion urine have been reported [59]. The use of GC-FID instead of GC-MS to analyze cheetah MF may have resulted in the absence of aldehydes and ketones found previously in tigers and leopards [41]. The use of gel electrophoresis made it difficult to identify cauxin in the following big cats: Asiatic lions (*Panthera leo persica)*; Sumatran tigers (*Panthera tigirs sumatrae)*; Persian leopards (*Panthera pardus saxicolor)*; jaguar (*Panthera onca)*; and clouded leopard (*Neofelis nebulosa*) because of its similar mass to urinary serum albumin [62].

To date, there is no published research on domestic or wild cats linking a chemical with specific odors associated with their scent marks. Thus, there is clearly a need to define characteristic odors by identifying key chemical constituents responsible for odor in a more reliable approach using analytical tools. Several studies have established the importance of odor in scent mark detection and signalling in domestic cats [161,165,206–208]. Scent marks contain specific chemicals which signal to receiving animals an odor message about age, strength, dominance, relatedness, and reproductive status [5,207]. The actual amount of time it takes to quantifiably determine differences in semiochemical composition of tigers is unknown, but it has been estimated that by human nose, a general decrease in detection has been noted after a period of two weeks [166].

## 4. Conclusions/Outlook

Chemical and sensory analyses of semiochemicals can potentially aid wildlife conservation. These volatile compounds are essential to the comprehension of animal communication. Large mammal scent-markings are of particular interest because they have not been studied in as much depth as insects and small mammals (e.g., rodents). Great cats, specifically, are facing complete eradication and could benefit from alternative and improved conservation approaches. Scent-marking sample and analytical techniques have their pitfalls and advantages, but have evolved in efficiency over the last decade. The most frequently implemented analytical techniques for characterizing scent marks of wildlife are: GC [55], GC-MS [44,56–59], GC-FID [31,44], GC-TOF-MS, nano-LC-MS [40], MALDI-TOF-MS [42,61,62], ESI-MS/MS [62], gel electrophoresis [62], TLC [31,33], GLC [31], and ESI-MS/MS [62].

Understanding of scent-marking constituency aids in the identification of key chemical markers responsible for behavior associated with mating, territoriality, and resource management. Without the input of sensory analysis, the last two steps in the understanding of ethochemistry cannot be executed. The use of animals, human olfaction, and simple GC analysis in the determination of odor composition is limiting at best. The implementation of MD-GC-MS-O, E-noses, and EOGs can help to bridge the knowledge gap about total odor composition of scent marks. This new found information can lead to wildlife management improvement and protection of large mammals and other groups of endangered species.

## Figures and Tables

**Table 1.** Summary of findings and knowledge gaps in the area of sample preparation and analysis techniques used to analyze large mammal scent-markings.

**Sample Preparation Technique**	**Chemical Analysis**	**Species**	**Type of Marking**	**Major Findings**	**Identified Needs/Gaps of Knowledge**
Solvent-based extraction [62]	MALDI-ToF MS; ESI-MS; ESI-MS/MS [62]	Lion *(P. leo persica);* Tiger *(P. tigris sumatrae);* Persian Leopard *(P. pardus saxicolor);* Snow leopard *(P. uncia);* Clouded leopard *(N. nebulosa)*	Urine	Cauxin was present in the urine of male cats; Intensity of cauxin in big cats was lower than domestic cats; Sequence in serum albumin signifies the relatedness of cat species; Felinine and its degradation products are putative pheromones	The exact role of cauxin as a catalyst in the conversion of dipeptide 3-methylbutanol-cysteinylglycine to glycine and felinine
Solvent-based extraction [41] SPME [124]	GC-FID, TLC [41] GC-MS [124]	Cheetah *(Acinonyx jubatus)*	Marking Fluid, Urine	3.87 ± 0.58 mg/ml total lipid extracted from cheetah MF; Composed of free fatty acids; Lipids have limited fixative property; Pantolactone found in urine	Development of analytical techniques should be performed for chemical i.d. of total marking fluid composition
Solvent-based extraction [118]	GC-MS [118]	Blackbuck *(Antelope cervicapra)*	Urine	28 major constituents were identified in the urine of all males; Three compounds were seen only in dominant males during the dominance hierarchy period	Functional role of compounds is needed to determine the role of compounds in social communication
SPME [124]	GC-MS, GC-PFPD, GC-FID [124]	African wild dog *(Lycaon pictus)*	Urine, Feces, Anal glands, Preputial glands	103 organic compounds detected; Squalene is a major component of urine, feces, anal gland; 11 compounds were species specific	Analytical methods not efficient in determination of chirality of identified compounds or positions of double bonds in unsaturated acids
Solvent-based extraction [107,125,126]	GC [107], GLC-FID [125,126]	Black-tailed deer *(Odocoileus hemionus columbians)*	Interdigital scent, Tarsal scent	Tarsal gland plays a role in sexual isolation between deer subspecies; 5 unsaturated lactones elicit licking behavior, excitement	Identification of specific odor profiles of the scent marks responsible for eliciting behaviors using GC
Solvent-based extraction [113,127] SPME [114]	GC–MS [113,114], HPLC [127]	Giant panda *(Ailuropoda melanoleuca)*	Anogenital gland secretions, Urine, Feces, Blood serum	Anogenital secretions composed of steroids, fatty acids, aldehydes, alkanes, alkenes, amines, terpenes, and furans; Glucocorticoid hormonal levels rise during mating season	Behavioral bioassay is needed to unveil how these compounds mediate synchronization of breeding
Solvent-based extraction [128]; Headspace sampling [18]	GC-MS [128], GC [128]	White-tailed deer *(Odocoileus virginianus)*	Tarsal scent	Characterized 63 compounds in females and 55 in males; Alcohols, aldehydes, alkanes, alkenes, amines, ethers, furans, and ketones occurred in the urine of either sex	Additional chemical analyses and behavioral bioassays for screening of biologically important compounds
Solvent-based extraction [31,33,129]; SEP [47]	GC-MS [33,47], GC [47], TLC [31], GLC [31,33,129], GC-FID [31,129]	Bengal tiger *(Panthera tigris tigris)*	Marking Fluid, Urine	Average lipid content of MF is 1.88 ± 0.75 mg/moL; 98 volatile compounds confirmed including ketones, fatty acids, lactones	Quantitative derivatization of major unsaturated compounds; Confirmation of 2-Acetyl-1-pyrroline for odor characterization
Solvent-based extraction [130]; Headspace autosampling [59] SPME[124]	GC-MS [59,124,130]	Lion *(Panthera leo)*	Marking Fluid, Urine	55 compounds i.d. and 7 are potentially species specific; Males' markings more similar than females; Males have higher levels of 2-butanone and females have higher concentrations of acetone; Pantolactone found in urine	Only samples with lipid confirmation were analyzed for composition, limiting the results
Solvent-based extraction [123,131], SPME [112], SPDE [45]	GC-MS [45,123], GC [112], GC-FID [131]	Strepsirrhine families	Urine	Acetone, 2-hexanone, 4-heptanone and 2-heptanone have a primal role in communication	Relationship between social and solitary species scent-markings; Quantitative differences between scent-markings of lemurs between seasons
Solvent-based extraction [132], Headspace sampling [132–134]	GC-MS [132,133], GC-FID [133], GC-FTLR [133], Reversed-phase HPLC[133]	Gray wolf *(Canis lupus)*	Feces, Urine	77 compounds in feces of adult wolves; Aromatic organic compounds, steroids, carboxylic acids, aldehydes, alcohols, squalene and α-tocopherol	Understanding of variations in chemicals related to sex, reproductive season, or social status
Solvent-based extraction [106,117], No-treatment [106,117]	GC-MS [106,117]	Koala *(Phascolarctos cinereus)*	Sternal gland secretion	Volatile fatty acids, aldehydes, ketones, mono- and sesquiterpenes were identified; Some volatile nitriles and oximes i.d. never determined in any mammalian skin gland	Incorporation of scent and chemical analysis to understand influence of age on marking detection and composition
Solvent-based extraction, micro-preparative GC [135]	GC-MS, GC-FID [135]	Brown-mantled tamarin *(Saguinus fusciollis)*	Scent mark	17 compounds responsible for the composition of marmoset scent-markings; 3 dienes, 1 squalene, 8 monoenes, 5 saturated compounds	Compounds at 0.01% concentration were omitted from analysis, possibly affecting the true total composition
Solvent-based extraction [116], Headspace extraction [105], SPME [55], SPE [42,55,136]	Radioimmuno Assay [105], GC-FID [105], GC-MS [105,116], GC [55], MALDI/TOF-MS [42], PAGE/electroblotting [42], MRS [116]	Asian elephant *(Elephas maximus)*	Urine	Combined headspace SPME and GC-MS determined 5alpha-androst-2-en-17beta-ol and -17-one to determine start of estrous and predict the period of parturition; 5 -androst-3 -ol-17-one and probably 5 -androst-3 -ol-17 -ol are generated from sulfate conjugates by a thermal process; Follicular LH2 identified as a preovulatory hormone in female elephants	Influences of environmental, hormonal, and genetic factors of musth are unknown
Solvent-based extraction [46,137], SPME [46,137,138], SFE [139], SDE [139], SWE [139]	GC[138], GC-MS [46,137–139], GC x GC, GC-MS-O [138]	Human *(Homo sapiens)*	Urine, Feces, Sweat, hand scent	The use of NaCl and KCl improved the extraction efficiencies of VOCs from urine, with NaCl being optimal	Additional qualitative and quantitative comparison of VOC profiles of multiple specimen samples collected simultaneously from individuals
Solvent-based [140]	GC, GC-MS, NMR [140]	American beaver *(Castor Canadensis)*	Castor sacs	5 phenolic compounds identified; 15 phenolic compounds previously identified in prior studies	Detection methods may have prohibited the confirmation of 10 phenolic compounds previously detected with TLC
SPME [124,141]	GC-MS [124,141]	Spotted hyena *(Crocuta crocuta)*	Feces	252 volatile compounds detected; Composition of scent marks indicate social status; Pantolactone found in feces	Use of GC-MS to measure the energy cost associated w/specific compounds in scent marks
SPDE, SPME [44] CHS, IFE [142]	GC-FID, GC-MS [44,142]	African elephant *(Loxodonta africana)*	Urine	Frontalin pheromone was found in elephant urine; endo- and exo-brevicomin, similar to frontalin, are also beetle pheromones; IFE and CHS headspace methods were equally significantly effective in detecting ketones and acids	Continued investigation of optimal extraction method for chiral columns
Precolumn heater technique [101,102]	GC-MS [101,102]	Reindeer *(Rangifer tarandus)*	Tarsal scent gland, Interdigital gland	Two of the major constituents have been identified as 1-hydroxy-7-methyl-3-octanone and 7-methyl-1-octen-3-one	Relationship between season and scent- marking concentrations
Precolumn heater technique [143]	GC-MS [143]	Bobcat *(Lynx rufus)*	Urine	Identified sulfide, disulfide, and trisulfide compounds	Further field studies on the role of dichloromethane in urine as an animal deterrent
Acid/steam distillation [144]	GC-MS [144]	Horse *(Equine caballus)*	Urine, feces, urine-marked feces	Fatty acids, alcohols, aldehydes, phenols, amines alkanes, tetradecanoic and hexadecanoic acids in feces differed based on maturity, sex, and reproductive stage	Lack of Chemosensory analyses could suggest role of marking cresol by stallions in masking mare feces odor.

* *Abbreviations*: GC/FTIR- gas chromatography/Fourier-transform infrared spectroscopy; RT-retention time, MALDI-TOF-MS matrix-assisted laser desorption ionization time of flight mass spectrometry; ESI-MS- electrospray ionization mass spectrometry; ESI-MS/MS-tandem mass spectrometry; GC-gas chromatography; VOC-volatile organic compounds; SPDE-solid phase dynamic extraction; AC-PDMS- activated charcoal (Carboxen)-polydimethylsiloxane; GLC-gas liquid chromatography, MRS-magnetic resonance spectroscopy; SEP-sample enrichment probe; SDE-simultaneous distillation-extraction; SWE-subcritical water extraction; SFE-supercritical fluid extraction; NMR-nuclear magnetic resonance; GC-PFPD-gas chromatography-pulsed flame photometric detector; CHS-contained headspace; IFE-Inverted funnel extraction; LH2-leutenizing hormone in luteal urine.

**Table 2.** Summary of simultaneous sensory and chemical analysis of scent-markings from endangered large mammals.

**Species**	**Aim**	**Type of Marking/Sample**	**Chemical/Sensory Analysis**	**Findings**	**Identified Needs/Gaps of Knowledge**	**Advantages/Disadvantages**
*Lemur catta* [131]	Demonstrate individual recognition of female genital marking in *Lemur catta*	Genital marking	GC-FID, Lemur olfaction	Only females have recognizable scent-markings	Further experiments on the occurrence of individual recognition	**Dis**- Animals showed a high variability in their motivation to investigate markings
*Elephas maximus* [44,146]	Review the response behavior by elephants to interpret chemical detection and ratio of enantiomers of frontalin based on sex, age, and stage of musth	Musth, Urine	GC-MS, Elephant olfaction	Compounds in urine and musth responsible for transport and behavior; Musth varies w/age and stage of Musth and/or frolatin component; Chirality in pheromones	Lack of information on pheromone variation over time of year and region; The interactions of pheromones with receptor proteins	**Adv**- SPE unlike headspace analysis, does not require the solute to be volatile to be extracted; **Dis-**Sample size of 6 males
*Homo Sapiens* [138,150]	Summarize the current knowledge on chemical and clinical aspects of body-derived VOCs.	Sweat, Urine, Feces, Breath	GC, GC-MS, GC x GC, GC-MS-O, E-noses	VOCs emitted from the body vary with age, diet, sex, physiological status and genetics	Minimal research on VOC diagnostic criteria for disease	**Adv**-GC-MS-O identified characteristic odorous VOCs that are in low abundance in various biological samples
Various Vertebrate and Invertebrate Species [151]	Review the history and developments in the area of olfactory biosensors that detect volatile compounds	Sub-tissue, Whole organisms	EOG, E-noses, SPR, FRET, SAW, FET, QCM	The ability to detect volatile compounds w/the same specificity as nature's olfactory machinery is applicable in environmental studies	SWCNT-based platforms will aid in developing a portable apparatus for olfaction in 10yrs	**Adv-** ORs in biosensors are more sensitive detectors of ligands than GC-MS and chemical “noses”; E-noses are real-time methods; **Dis-** EOG provides no information about or molecular basis of olfaction w/o molecular analysis; Luminescence optical assays have low detection limits; E-noses lack biorecognition stability and portability

* *Abbreviations*: LC=Liquid Chromatography, GC=Gas Chromatography, VNO=Vomeral Nasal Organ, FID Flame Ionization Detector, GC-MS-O=Gas Chromatography-Mass Spectrometry-Olfactometry, EOG=Electro-olfactogram, OR=Olfactory Receptor, SPR=Surface Plasmon Resonance, FET=Field-effect Transistors, SAW=Surface Acoustic Wave, FRET=Förster resonance energy transfer, QCM=Quartz Crystal Microbalance, SWCNT=single-wall carbon nanotube

**Table 3.** Number/percentage of articles that focus on categorizing scent-marking behaviors in wild cats and their relationships to conservation.

**Species**	**Behaviors Associated with Scent-Marking**				**Relationship to Conservation**
	**Reproduction**	**Territoriality**	**Dominance**	**Other**	
Tiger (*Panthera tigris*)	(5) 23.8% [31,33,130,163,166,167]	(4) 19.04% [130,163,166,168]	(4) 19.04% [130, 169–171]	(8) 38.09% [62,68,129, 172–176]	-Implement better wildlife management practices-Provide adequate land and resources-Increase lifespan of captive and wild tigers-Determine populations-Understand chemosignalling-Indicator of reproductive status, territory, and physical condition
Lion (*Panthera leo*)	(1) 9.09% [59]	(3) 27.27% [163,177,178]	(3) (27.27%) [170,171,179]	(4) 36.36% [62,174,175,180]	-Taxonomical separation and classification-Sex and identification-Understand chemosignalling
Puma (*Puma concolor*)	(2) 18.18% [181,182]	(6) 54.54% [70,183–187]	(1) 9.09% [70]	(2) 18.18% [174,185]	-Population assessments-Territoriality-Phylogenetic reconstruction
Snow leopards (*Panthera uncia)*	(2) 25.00% [154,184]	(3) 37.50% [154,164,186]	(0) 0.00%	(3) 37.50% [174,188,189]	-Population estimates-Phylogenetic reconstruction-Distribution
Cheetah (*Acinonyx jubatus*)	(1) 16.67% [41,190]	(2) 33.34% [41,191]	(1) 16.67% [41]	(2) 33.34% [41,174]	-Marking fluid is an indicator of physical condition-Population estimates
Kalahari leopards *(Panthera pardus)*	(2) 25.00% [165,192]	(3) 37.50% [192,193]	(1) 12.50% [192]	(2) 25.00% [174,194]	-Population assessments-Territoriality-Phylogenetic classification-Diet

## References

[1] Campbell-Palmer R., Rosell F. (2011). The importance of chemical communication studies to mammalian conservation biology: A review. Biol. Conserv..

[2] Forrester G.S. (2008). A multidimensional approach to investigations of behaviour: revealing structure in animal communication signals. Anim. Behav..

[3] Sunquist M., Sunquist F. (2002). Wild Cats of the World.

[4] Bullock T.H. (1999). The future of research on electroreception and electrocommunication. J. Exp. Biol..

[5] Gosling L.M., Roberts S.C., Slater P.J.B., Rosenblatt J.S., Snowdon C.T., Roper T.J. (2001). Scent-marking by male mammals: Cheat-proof signals to competitors and mates. Advances in the Study of Behavior.

[6] Schulz S., Burger B. (2005). Mammalian Semiochemicals. The Chemistry of Pheromones and Other Semiochemicals II.

[7] Happ G. (1973). Chemical Signals Between Animals: Allomones and Pheromones. Humoral Control of Growth and Differentiation.

[8] Wyatt T.D. (2003). Pheromones and Animal Behaviour.

[9] Apps P. (2013). Are mammal olfactory signals hiding right under our noses?. Naturwissenschaften.

[10] Brahmachary R.L. (1986). Ecology and chemistry of mammalian pheromones. Endeavour.

[11] Alberts A. (1992). Constraints on the design of chemical communication systems in terrestrial vertebrates. Am. Nat..

[12] Dicke M., Sabelis M.W. (1988). Infochemical terminology: Based on cost-benefit analysis rather than origin of compounds?. Funct. Ecol..

[13] Touhara K., Vosshall L.B. (2009). Sensing odorants and pheromones with chemosensory receptors. Annu. Rev. Physiol..

[14] Albone E.S. (1984). Mammalian Semiochemistry: The Investigation of Chemical Signals Between Mammals.

[15] Pageat P., Gaultier E. (2003). Current research in canine and feline pheromones. Vet. Clin. North Am. Small Anim. Pract..

[16] Kitchen D.W. (1974). Social behavior and ecology of the pronghorn. Wildlife Monogr..

[17] Wood W.F. (2011). 2-Ethyl-3methylpyrazine in the subauricular and median glands of pronghorn. Antilopcapra americana. Biochem. Sys. Ecol..

[18] Gassett J.W., Wiesler D.P., Baker A.G., Osborn D.A., Miller K.V., Marchinton R.L., Novotny M. (1996). Volatile compounds from interdigital gland of male white-tailed deer (*Odocoileus virginianus*). J. Chem. Ecol..

[19] Bossert W.H., Wilson E.O. (1963). The analysis of olfactory communication among animals. J.Theor. Biol..

[20] Atkins M.D. (1980). Introduction to Insect Behaviour.

[21] Chamero P., Marton T.F., Logan D.W., Flanagan K., Cruz J.R., Saghatelian A., Cravatt B.F., Stowers L. (2007). Identification of protein pheromones that promote aggressive behaviour. Nature.

[22] Gleason K., Reynierse J. (1969). The behavioral significance of pheromones in vertebrates. Psychol. Bull..

[23] Bradbury J.W., Vehrencamp S.L. (2011). Principles of Animal Communication.

[24] Apps P.J., Viljoen H.W., Richardson P.R.K., Pretorius V. (1989). Volatile components of anal gland secretion of aardwolf (*Proteles cristatus*). J. Chem. Ecol.

[25] Maynard Smith J. (1982). The Evolution and the Theory of Games.

[26] Parker G., Rubenstein D. (1981). Role assessment, reserve strategy, and acquisition of information in asymmetric animal conflicts. Anim. Behav..

[27] Yamazaki K., Boyse E., Mike V., Thaler H., Mathieson B., Abott J., Boyse J., Zayas Z., Thomas L. (1976). Control of mating preferences in mice by genes in the major histocompatibility complex. J. Exp. Med.

[28] Liberles S.D. (2009). Trace Amine-associated Receptors Are Olfactory Receptors in Vertebrates. Ann. N. Y. Acad. Sci..

[29] Amoore J.E. (1963). Stereochemical Receptor Theory. Nature.

[30] Bossert W.H., Wilson E.O. (1963). The analysis of olfactory communication among animals. J. Theoretical Biol..

[31] Poddar-Sarkar M. (1996). The fixative lipid of tiger pheromone. J. Lipid Mediat. Cell. Signal..

[32] Albone E.S., Grönnerberg T.O. (1977). Lipids of the anal sac secretions of the red fox, *Vulpes vulpes* and of the lion. Panthera leo. J. Lipid Res..

[33] Brahmachary R., Poddar-Sarkar M., Dutta J. (1990). The aroma of rice…and tiger. Nature.

[34] Pawliszyn J. (1997). Solid Phase Microextraction: Theory and Practice.

[35] Eke Z., Torkos K. (2012). Sample Preparation for Gas Chromatography. Encyclopedia of Analytical Chemistry.

[36] Drea C.M., Boulet M., Delbarco-Trillo J., Greene L.K., Sacha C.R., Goodwin T.E., Dubay G.R. (2013). The “Secret” in secretions: Methodological considerations in deciphering primate olfactory communication. Am. J. Primatol..

[37] de Kooning S., Janssen H.-G., Brinkman U.A.T. (2009). Modern methods of sample preparation for GC analysis. Chromatographia.

[38] Augusto F., Luiz Pires Valente A. (2002). Applications of solid-phase microextraction to chemical analysis of live biological samples. Trend. Anal. Chem..

[39] Bligh E., Dyer W. (1959). A rapid method of total lipid extraction and purification. Can. J. Biochem. Physiol..

[40] Papes F., Logan D.W., Stowers L. (2010). The vomeronasal organ mediates interspecies defensive behaviors through detection of protein pheromone homologs. Cell.

[41] Poddar-Sarkar M., Brahmachary R.L. (1997). Putative semiochemicals in the African cheetah (*Acinonyx jubatus*). J. Lipid Med. Cell Signal..

[42] Lazar J., Greenwood D.R., Rasmussen L., Bang I., Prestwich G. (2004). Elephant albumin: A multipurpose pheromone shuttle. Chem. Biol..

[43] Tholey A., Gluckmann M., Seemann K., Karas M. (2008). Proteomics Sample Preparation.

[44] Goodwin T., Eggert M., House S., Weddell M., Schulte B., Rasmussen L.E.L. (2006). Insect pheromones and precursors in female African elephant urine. J. Chem. Ecol..

[45] Delbarco-Trillo J., Burkert B.A., Goodwin T.E., Drea C.M. (2011). Night and day: The comparative study of strepsirrhine primates reveals socioecological and phylogenetic patterns in olfactory signals. J. Evol. Biol..

[46] Kusano M., Mendez E., Furton K.G. (2011). Development of headspace SPME method for analysis of volatile organic compounds present in human biological specimens. Anal. Bioanal. Chem..

[47] Burger B.V., Viviers M.Z., Bekker J.P.I., le Roux M., Fish N., Fourie W.B., Weibchen G. (2008). Chemical characterization of territorial marking fluid of male Bengal tiger. Panthera tigris. J. Chem. Ecol..

[48] Fustinoni S., Giampiccolo R., Pulvirenti S., Buratti M., Colombi A. (1999). Headspace solid-phase microextraction for the determination of benzene, toluene, ethylbenzene and xylenes in urine. J. Chromatogr. B Biomed. Sci. App..

[49] Cudjoe E., Wiederkehr T.B., Brindle I.D. (2005). Headspace gas chromatography-mass spectrometry: A fast approach to the identification and determination of 2-akyl-3-methoxypyrazine pheromones in lady bugs. Analyst.

[50] de Koning S., Janssen H.-G. (2009). Modern Methods of Sample Preparation for GC Analysis. Chromatographia.

[51] Cai L., Koziel J.A., O'Neal M.E. (2007). Determination of characteristic odorants from Harmonia axyridis beetles using *in vivo* solid-phase microextraction and multidimensional gas chromatography–mass spectrometry–olfactometry. J. Chrom. A.

[52] Spinhirne J.P., Koziel J.A., Chirase N. (2003). A device for noninvasive on-site sampling of cattle breath with solid phase microextraction. Biosyst. Eng..

[53] Spinhirne J.P., Koziel J.A., Chirase N. (2004). Sampling and analysis of VOCs in bovine breath using solid-phase microextraction and gas chromatography-mass spectrometry. J. Chrom. A.

[54] Skoog D., Holler F.J., Crouch S.R. (2006). Principles of Instrumental Analysis.

[55] Greenwood D.R., Comeskey D., Hunt M.B., Rasmussen L.E.L. (2005). Chemical communication: Chirality in elephant pheromones. Nature.

[56] Wang Y., Hossain D., Perry P.L., Adams B., Lin J. (2012). Characterization of volatile and aroma-impact compounds in persimmon (Diospyros kaki L., var. Triumph) fruit by GC-MS and GC-O analyses. Flavour Frag. J..

[57] Dehnhard M., Hatt J.M., Eulenberger K., Ochs A., Strauss G. (2003). Headspace solid-phase microextraction (SPME) and gas chromatography–mass spectrometry (GC–MS) for the determination of 5α-androst-2-en-17-one and -17β-ol in the female Asian elephant: Application for reproductive monitoring and prediction of parturition. J. Steroid Biochem. Mol. Biol..

[58] Archunan G., Rajagopal T. (2013). Detection of estrus in Indian blackbuck: Behavioural, hormonal and urinary volatiles evaluation. Gen. Comp. Endocr..

[59] Andersen K.F., Vulpius T. (1999). Urinary Volatile Constituents of the Lion. Panthera leo. Chem. Senses.

[60] Lynn M., Jane H., Christopher G., John L., Robert B. (2007). Characterization of cauxin in the urine of domestic and big cats. J. Chem. Ecol..

[61] Miyazaki M., Yamashita T., Suzuki Y., Saito Y., Soeta S., Taira H., Suzuki A. (2006). A major urinary protein of the domestic cat regulates the production of felinine, a putative pheromone precursor. Chem. Biol..

[62] McLean L., Hurst J., Gaskell C., Lewis J.M., Beynon R. (2007). Characterization of cauxin in the urine of domestic and big cats. J. Chem. Ecol..

[63] Chai M., Pawliszyn J. (1995). Analysis of environmental air samples by solid-phase microextraction and gas chromatography/ion trap mass spectrometry. Environ. Sci. Technol..

[64] Osada K., Tashiro T., Mori K., Izumi H. (2008). The identification of attractive volatiles in aged male mouse urine. Chem. Senses.

[65] Osada K., Yamazaki K., Curran M., Bard J., Smith B.P.C., Beauchamp G.K. (2003). The scent of age. P. Roy. Soc. B-Biol. Sci..

[66] Johnston R.E., Schmidt T. (1979). Responses of hamsters to scent marks of different ages. Behav. Neural Biol..

[67] Seidensticker J. (1997). Saving the Tiger. Wildlife Soc. B..

[68] Kerley L.L., Salkina G.P. (2007). Using scent-matching dogs to identify individual Amur tigers from scats. J. Wildl. Manage..

[69] McBride R.T., McBride R.T., McBride R.M., McBride C.E. (2008). Counting pumas by categorizing physical evidence. Southeast. Nat..

[70] Harmsen B.J., Foster R.J., Gutierrez S.M., Marin S.Y., Doncaster C.P. (2010). Scrape-marking behavior of jaguars (*Panthera onca*) and pumas (*Puma concolor*). J. Mammal..

[71] Rock F., Barsan N., Weimar U. (2008). Electronic nose: Current status and future trends. Chem. Rev..

[72] Mugford R.A.a., N. N.W (1970). Pheromones and their effect on aggression in mice. Nat. (Lond.).

[73] Roberts S.C., Gosling L.M. (2004). Manipulation of olfactory signaling and mate choice for conservation breeding: a case study of harvest mice. Conserv. Biol.

[74] Wasser S.K., Davenport B., Ramage E.R., Hunt K.E., Parker M., Clarke C., Stenhouse G. (2004). Scat detection dogs in wildlife research and management: Application to grizzly and black bears in the Yellowhead Ecosystem, Alberta, Canada. Can. J. Zool..

[75] Burger B.V., Nell A.E., Spies H.S.C., Le Roux M., Bigalke R.C., Brand P.A.J. (1999). Mammalian exocrine secretions. XII: Constituents of interdigital secretions of bontebok, Damaliscus dorcas dorcas, and blesbok, D. d. phillipsi. J. Chem. Ecol..

[76] Sorensen P.W., Hoye T.R. (2010). Pheromones in Vertebrates. Comprehensive Natural Products II Chemistry and Biology: Pheromones in Vertebrates.

[77] Porter R.H. (1999). Olfaction and human kin recognition. Genetica.

[78] Gopel W. (1998). Chemical imaging: I. Concepts and visions for electronic and bioelectronic noses. Sens. Actuat..

[79] Pearce T.C. (1997). Computational parallels between the biological olfactory pathway and its analogue ‘The Electronic Nose’: Part II. Sensor-based machine olfaction. J. Biosyst..

[80] Grégoire L., Marie-Annick P., Denise G., Jean-Jacques R., Roland S., Edith P.A. (2003). Ligand-specific dose–response of heterologously expressed olfactory receptors. Eur. J. Biochem..

[81] Malnic B., Hirono J., Sato T., Buck L. (1999). Combinatorial receptor codes for odors. Cell.

[82] Lee J.Y., Ko H.J., Lee S.H., Park T.H. (2006). Cell-based measurement of odorant molecules using surface plasmon resonance. Enzyme Microb. Tech..

[83] Ko H.J., Park T.H. (2008). Enhancement of odorant detection sensitivity by the expression of odorant-binding protein. Biosens. Bioelectron..

[84] Hou Y., Jaffrezic-Renault N., Martelet C., Zhang A., Minic-Vidic J., Gorojankina T., Persuy M.A., Pajot-Augy E., Salesse R., Akimov V. (2007). A novel detection strategy for odorant molecules based on controlled bioengineering of rat olfactory receptor I7. Biosens. Bioelectron..

[85] Ko H.J., Park T.H. (2005). Piezoelectric olfactory biosensor: ligand specificity and dose-dependence of an olfactory receptor expressed in a heterologous cell system. Biosens. Bioelectron..

[86] Mitsubayashi K., Hashimoto Y. (2002). Bioelectronic sniffer device for trimethylamine vapor using flavin containing monooxygenase. IEEE Sens. J..

[87] Mitsubayashi K., Nishio G., Sawai M., Saito T., Kudo H., Saito H., Otsuka K., Noguer T., Marty J.-L. (2008). A bio-sniffer stick with FALDH (formaldehyde dehydrogenase) for convenient analysis of gaseous formaldehyde. Sens. Actuator B-Chem..

[88] Laor J., Koziel J.A., Cai L., Ravid U. (2008). Enhanced characterization of dairy manure odor by time-increased headspace solid phase microextraction and multidimensional gas chromatography–mass spectrometry-olfactometry. J. Air Waste Manage..

[89] Lo Y.C., Koziel J.A., Cai L., Hoff S.J., Jenks W.S., Xin H. (2008). Simultaneous chemical and sensory characterization of VOCs and semi-VOCs emitted from swine manure using SPME and multidimensional gas chromatography-mass spectrometry-olfactometry system. J. Environ. Qual..

[90] Wright D.W., Eaton D.K., Nielsen L.T., Kuhrt F.W., Koziel J.A., Spinhirne J.P., Parker D.B. (2005). Multidimensional gas chromatography−olfactometry for the identification and prioritization of malodors from confined animal feeding operations. J. Agri. Food Chem..

[91] Bulliner E.A., Koziel J.A., Cai L., Wright D. (2006). Characterization of livestock odors using steel plates, solid-phase microextraction, and multidimensional gas chromatography–mass spectrometry–olfactometry. J. Air Waste Manage..

[92] Zhang S., Cai L., Koziel J.A., Hoff S.J., Schmidt D.R., Clanton C.J., Jacobson L.D., Parker D.B., Heber A.J. (2010). Field air sampling and simultaneous chemical and sensory analysis of livestock odorants with sorbent tubes and GC–MS/olfactometry. Sens. Actuat. B-Chem..

[93] Koziel J.A., Lo Y.M., Cai L., Wright D. (2010). Simultaneous characterization of VOCs and livestock odors using solid-phase microextraction-gas chromatography-mass spectrometry-olfactometry. Chem. Eng. Trans..

[94] Cai L., Koziel J.A., Davis J., Lo Y.C., Xin H. (2006). Characterization of volatile organic compounds and odors by *in-vivo* sampling of beef cattle rumen gas, by using solid phase microextraction and gas chromatography-mass spectrometry-olfactometry. Anal. Bioanal. Chem..

[95] Cai L., Koziel J.A., Liang Y., Nguyen A.T., Xin H. (2007). Evaluation of zeolite for control of odorants emissions from simulated poultry manure storage. J. Environ. Qual..

[96] Cai L., Koziel J.A., Dharmadhikari M., van Leeuwen J. (2009). Rapid determination of trans-resveratrol in red wine by solid-phase microextraction with on-fiber derivatization and multidimensional gas chromatography–mass spectrometry. J. Chrom. A.

[97] Koziel J.A., Lo Y.-C., Wright D., Trabue S., Kerr B. (2005). The use of SPME and multidimensional GC-MS-Olfactometry system for identification of key odorants from swine manure.

[98] Koziel J.A., Cai L., Wright D., Hoff S.J. (2006). Solid-phase microextraction as a novel air sampling technology for improved, GC-olfactometry-based assessment of livestock odors. J Chromatogr Sci..

[99] Hollmann M., Boertz J., Dopp E., Hippler J., Hirner A.V. (2010). Parallel on-line detection of a methylbismuth species by hyphenated GC/EI-MS/ICP-MS technique as evidence for bismuth methylation by human hepatic cells. Metallomics.

[100] Ramos L. (2011). Critical overview of selected contemporary sample preparation techniques. J. Chrom. A.

[101] Andersson G., Brundin A., Andersson K. (1979). Volatile compounds from the interdigital gland of reindeer (*Rangifer t. tarandus L.*). J. Chem. Ecol..

[102] Andersson G., Andersson K., Brundin A., Rappe C. (1975). Volatile compounds from the tarsal scent gland of reindeer (*Rangifer tarandus*). J. Chem. Ecol..

[103] Moors M., Massart D.L., McDowall R.D. (1994). Analyte isolation by solid phase extraction (SPE) on silica-bonded phases: Classification and recommended practices. J. Pure Appl. Sci..

[104] East M.L., Dehnhard M., Goodwin T., Songsasen N., Broederdorf L., Burkert B., Chen C.J., Jackson S., Keplinger K.B., Rountree M. (2013). Hemiterpenoids and Pyrazines in the Odoriferous Urine of the Maned Wolf (Chrysocyon brachyurus). Chemical Signals in Vertebrates.

[105] Rasmussen L.E.L., Perrin T.E. (1999). Physiological correlates of musth: Lipid metabolites and chemical composition of exudates. Physiol. Behav..

[106] Salamon M., Davies N.W. (1998). Identification and variation of volatile compounds in sternal gland secretions of male koalas (*Phascolarctos cinereus*). J. Chem. Ecol..

[107] Brownlee R., Silverstein R., Müller-Schwarze D., Singer A. (1969). Isolation, identification, and function of the chief component of male tarsal scent in black-tailed deer. Nature.

[108] Soini H., Bruce K., Wiesler D., David F., Sandra P., Novotny M. (2005). Stir bar sorptive extraction: A new quantitative and comprehensive sampling technique for determination of chemical signal profiles from biological media. J. Chem. Ecol..

[109] Baltussen E., Sandra P., David F., Cramers C. (1999). Stir bar sorptive extraction (SBSE), a novel extraction technique for aqueous samples: Theory and principles. J. Microcolumn Sep..

[110] Baltussen E., Cramers C., Sandra P. (2002). Sorptive sample preparation—A review. Anal. Bioanal. Chem..

[111] Pohorecky L.A., Blakley G.G., Ma E.W., Soini H.A., Wiesler D., Bruce K.E., Novotny M.V. (2008). Social housing influences the composition of volatile compounds in the preputial glands of male rats. Horm. Behav..

[112] Hayes R.A., Morelli T.L., Wright P.C. (2006). Volatile components of lemur scent secretions vary throughout the year. Am. J. Primatol..

[113] Yuan H., Liu D.Z., Sun L.X., Wei R.P., Zhang G.Q., Sun R.Y. (2004). Anogenital gland secretions code for sex and age in the giant panda. Ailuropoda melanoleuca. Can. J. Zool..

[114] Hagey L., MacDonald E. (2003). Chemical Cues Identify Gender and Individuality in Giant Pandas (*Ailuropoda melanoleuca*). J. Chem. Ecol..

[115] Kulkarni S.M. (2007). Sol-gel Immobilized Cyano-polydimethylsiloxane and Short Chain Polyethylene Glycol Coatings for Capillary Microextraction Coupled to Gas Chromatography.

[116] Rasmussen L.E.L., Lee T.D., Roelofs W.L., Zhang A., Daves G.D. (1996). Insect pheromone in elephants. Nature.

[117] Johnston R., Müller-Schwarze D., Sorensen P., Salamon M., Davies N., Stoddart D.M. (1999). Olfactory Communication in Australian Marsupials with Particular Reference to Brushtail Possum, Koala, and Eastern Grey Kangaroo. Advances in Chemical Signals in Vertebrates.

[118] Rajagopal T., Archunan G., Geraldine P., Balasundaram C. (2010). Assessment of dominance hierarchy through urine scent marking and its chemical constituents in male blackbuck *Antelope cervicapra*, a critically endangered species. Behav. Process..

[119] Biniecka M., Caroli S. (2011). Analytical methods for the quantification of volatile aromatic compounds. Trend. Anal. Chem..

[120] Barja L.D., Silván G., Illera J.C. (2008). Relationships between sexual and stress hormone levels in feces and marking behavior in a wild population of Iberian wolves (*Canis lupus signatus*). J. Chem. Ecol..

[121] Marriott P.J., Chin S.-T., Maikhunthod B., Schmarr H.-G., Bieri S. (2012). Multidimensional gas chromatography. Trend. Anal. Chem..

[122] Sasamoto K., Ochiai N. (2010). Selectable one-dimensional or two-dimensional gas chromatography-mass spectrometry with simultaneous olfactometry or element-specific detection. J. Chromatogr. A.

[123] Scordato E.S., Dubay G., Drea C.M. (2007). Chemical composition of scent marks in the ringtailed lemur (*Lemur catta*): Glandular differences, seasonal variation, and individual signatures. Chem. Senses.

[124] Apps P., Mmualefe L., McNutt J.W. (2012). Identification of volatiles from the secretions and excretions of African wild dogs (*Lycaon pictus*). J. Chem. Ecol..

[125] Beauchamp G.K., Doty R.L. (1976). The Pheromone Concept in Mammalian Chemical Communication: A Critique. Mammalian Olfaction, Reproductive Processes and Behaviour.

[126] Müller-Schwarze D. (1971). Pheromones in black-tailed deer (*Odocoileus heminonus columbianus*). Anim. Behav..

[127] Kersey D.C., Wildt D.E., Brown J.L., Huang Y., Snyder R.J., Monfort S.L. (2010). Parallel and seasonal changes in gonadal and adrenal hormones in male giant pandas (*Ailuropoda melanoleuca*). J. Mammal..

[128] Miller K.V., Jemiolo B., Gassett J.W., Jelinek I., Wiesler D., Novotny M. (1998). Putative chemical signals from white-tailed deer (*Odocoileus virginianus*): Social and seasonal effects on urinary volatile excretion in males. J. Chem. Ecol..

[129] Poddar-Sarkar M., Brahmachary R.L., Dutta J. (1991). Short Chain free fatty acid as a putative pheromone in the marking fluid of tiger. J. Indian Chem. Soc.

[130] Asa C.S. (1993). Relative contributions of urine and anal-sac secretions in scent marks of large felids. Am. Zool..

[131] Palagi E., Dapporto L. (2006). Beyond odor discrimination: Demonstrating individual recognition by scent in Lemur catta. Chem. Senses.

[132] Martín J., Barja I., López P. (2010). Chemical scent constituents in feces of wild Iberian wolves (*Canis lupus signatus*). Biochem. Sys. Ecol..

[133] Raymer J., Wiesler D., Novotny M., Asa C., Seal U.S., Mech L.D. (1984). Volatile constituents of wolf (*Canis lupus*) urine as related to gender and season. Experientia.

[134] Raymer J., Wiesler D., Novotny M., Asa C., Seal U.S., Mech L.D. (1986). Chemical scent constituents in urine of wolf (*Canis lupus*) and their dependence on reproductive hormones. J. Chem. Ecol..

[135] Smith A.B., Yarger R.G., Epple G. (1976). The major volatile constituents of the marmoset (*Saguinus fuscicollis*) scent mark. Tetrahedron Lett..

[136] Schulte B.A., Freeman E.W., Goodwin T.E., Hollister-Smith J., Rasmussen L.E.L. (2007). Honest signalling through chemicals by elephants with applications for care and conservation. Appl. Anim. Behav. Sci..

[137] Curran A.M., Ramirez C.F., Schoon A.A., Furton K.G. (2007). The frequency of occurrence and discriminatory power of compounds found in human scent across a population determined by SPME-GCMS. J. Chromat. B, Analyt. Technol. Biomed. Life Sci..

[138] Shirasu M., Touhara K. (2011). The scent of disease: Volatile organic compounds of the human body related to disease and disorder. J. Biochem..

[139] Prada P.A., Curran A.M., Furton K.G. (2010). Comparison of extraction methods for the removal of volatile organic compounds (VOCs) present in sorbents used for human scent evidence collection. J. Anal. Meth..

[140] Tang R., Webster F., Müller-Schwarze D. (1993). Phenolic compounds from male castoreum of the North American beaver,Castor canadensis. J. Chem. Ecol..

[141] Burgener N., Dehnhard M., Hofer H., East M.L. (2009). Does anal gland scent signal identity in the spotted hyaena?. Anim. Behav..

[142] Ganswindt A., Heistermann M., Hodges K. (2005). Physical, physiological, and behavioral correlates of musth in captive African elephants (*Loxodonta africana*). Physiol. Biochem. Zool..

[143] Mattina M.J.I., Pignatello J.J., Swihart R.K. (1991). Identification of volatile components of bobcat (*Lynx rufus*) urine. J. Chem. Ecol..

[144] Kimura R. (2001). Volatile substances in feces, urine and urine-marked feces of feral horses. Can. J. An. Sci..

[145] Valenta Z., Khaleque A., Rashid M.H. (1960). cis-cyclohexane-1,2-Diol in the Beaver Gland. Experentia.

[146] Rasmussen L.E.L. (1999). Evolution of chemical signals in the Asian elephant. *Elephas maximus*: behavioural and ecological influences. J. Biosci..

[147] Meyer J.M., Goodwin T.E., Schulte B.A. (2008). Intrasexual chemical communication and social responses of captive female African elephants. Loxodonta africana. Anim. Behav..

[148] Merte C.E., Goodwin T.E., Schulte B.A. (2010). Male and female developmental differences in chemosensory investigations by African elephants (*Loxodonta africana*) approaching waterholes. Behav. Ecol. Sociobiol..

[149] Goodwin T.E., Broederdorf L.J., Burkert B.A., Hirwa I.H., Mark D.B., Waldrip Z.J., Kopper R.A., Sutherland M.V., Freeman E.W., Hollister-Smith J.A. (2012). Chemical signals of elephant musth: temporal aspects of microbially-mediated modifications. J. Chem. Ecol..

[150] Friedrich M.J. (2009). Scientists seek to sniff out diseases. J.A.M.A..

[151] Glatz R., Bailey-Hill K. (2011). Mimicking nature's noses: From receptor deorphaning to olfactory biosensing. Prog. Neurobiol..

[152] Peters R.P., Mech L.P. (1978). Scent-marking in wolves. Am. Sci..

[153] East M.L., Dehnhard M., Hummel H. (2013). A Historical Perspective on the Identification of Substances in the Territorial Scent Marks of Male Klipspringer Antelope *Oreotragus oreotragus*. Chemical Signals in Vertebrates 12.

[154] Jackson R., Ahlborn G. (1989). Catching a ghost. J. Int. Wildl..

[155] Pazitna A., Janoskova N., Spanik I. (2013). Multidimensional gas chromatography and its applications in food and environmental analysis. Acta Chim. Slov..

[156] de Alencastro L.F., Grandjean D., Tarradellas J. (2003). Application of multidimensional (heart-cut) gas chromatography to the analysis of complex mixtures of organic pollutants in environmental samples. Chimia.

[157] Li X., Dai X., Yin X., Li M., Zhao Y., Zhou J., Huang T., Li H. (2013). Impurity analysis of pure aldrin using heart-cut multi-dimensional gas chromatography-mass spectrometry. J. Chromatogr. A.

[158] von Muhlen C., Khummueng W., Zini C.A., Caramao E.B. (2006). Detector technologies for comprehensive two-dimensional gas chromatography. J. Sep. Sci..

[159] Begnaud F., Chaintreau A. (2005). Multidimensional gas chromatography using a double cool-strand interface. J. Chromatogr. A.

[160] Marriott P.J., Eyres G.T., Dufour J.P., Wust C., Yeretzian C. (2009). Opportunities for Flavor Analysis Through Hyphenation.

[161] Mellen J.D. (1993). A comparative analysis of scent-marking, social and reproductive behavior in 20 species of small cats (*Felis*). Am. Zool..

[162] Knecht M., Hummel T. (2004). Recording of the human electro-olfactogram. Physiol. Behav..

[163] Poddar-Sarkar M., Chakroborty A., Bhar R., Brahmachary R.L. (2008). Putative pheromones of lion mane and its ultrastructure. Chem. Sig. Vert..

[164] Soini H., Linville S., Wiesler D., Posto A., Williams D., Novotny M. (2012). Investigation of scents on cheeks and foreheads of large felines in connection to the facial marking behavior. J. Chem. Ecol..

[165] du P. Bothma J., le Richet E.A.N. (1995). Evidence of the use of rubbing, scent-marking and scratching-posts by Kalahari leopards. J. Arid Environ..

[166] David Smith J.L., McDougal C., Miquelle D. (1989). Scent marking in free-ranging tigers. Panthera tigris. Anim. Behav..

[167] Brahmachary R.L. (1996). The expanding world of 2-acetyl-1-pyrroline. Curr. Sci..

[168] Sunquist M. (1981). The Social Organization of Tigers (Panthera tigris) in Royal Chitawan National Park, Nepal.

[169] Sugimoto T., Nagata J., Aramilev V., Belozor A., Higashi S., McCullough D. (2006). Species and sex identification from faecal samples of sympatric carnivores, Amur leopard and Siberian tiger, in the Russian Far East. Conserv. Genet..

[170] Apfelbach R., Blanchard C.D., Blanchard R.J., Hayes R.A., McGregor I.S. (2005). The effects of predator odors in mammalian prey species: A review of field and laboratory studies. Neurosci. Biobehav. Rev..

[171] Joseph S., Thomas A.P., Satheesh R., Sugathan R. (2007). Foraging ecology and relative abundance of large carnivores in Parambikulam Wildlife Sanctuary, southern India. Zoos Print J..

[172] Khan M.M.H., Chivers D.J. (2007). Habitat preferences of tigers *Panthera tigris* in the Sundarbans East Wildlife Sanctuary, Bangladesh, and management recommendations. Oryx.

[173] Spielman J.S. (2000). An Evaluation of the Function of Scent-marking in Carnivores with a Specific Study into the Effects of Pheromone Enrichment for Captive Tigers (Panthera tigris) and lions (Panthera leo).

[174] Bininda-Emonds O.R.P., Decker-Flum D.M., Gittleman J.L. (2001). The utility of chemical signals as phylogenetic characters: an example from the Felidae. Biol. J. Linn. Soc..

[175] Majumder P., Brahmachary R.L., Sarkar M., Dutta J. (1993). Evolution of Chemical Signals. Human Population Genetics.

[176] Gaultier E., Falewee C., Bougrat L., Pageat P., Mills D.S. (2005). The introduction of a female tiger (*Panthera tigris*) in a pre-established group of two neutered males: A case study. Current Issues and Research in Veterinary Behavioral Medicine.

[177] Lehmann M.B., Funston P., Owen C., Slotow R. (2008). Home range utilisation and territorial behavior of lions (*Panthera leo*) on Karongwe Game Reserve, South Africa. PLoS One.

[178] Mosser A., Packer C. (2009). Group territoriality and the benefits of sociality in the African lion. Panthera leo. Anim. Behav..

[179] Gittleman J., Gorman M., Trowbridge B. (1989). The Role of Odor in the Social Lives of Carnivores. Carnivore Behavior, Ecology, and Evolution.

[180] Kleiman D.G., Eisenberg J.F. (1973). Comparisons of canid and felid social systems from an evolutionary perspective. Anim. Behav..

[181] Pierce B.M., Vernon C.B., Chetkiewicz C.-L.B., Wehausen J.D. (1998). Timing of feeding bouts of mountain lions. J. Mammal..

[182] Brown J.L., Wasser S.K., Wildt D.E., Graham L.H. (1994). Comparative aspects of steroid-hormone metabolism and ovarian activity in felids, measured noninvasively in feces. Biol. Reprod..

[183] Villepique J.T., Pierce B.M., Bleich V.C., Bowyer R.T. (2011). Diet of cougars (*Puma concolor*) following a decline in a population of mule deer (*Odocoileus hemionus*): Lack of evidence for switching prey. Southwest. Nat..

[184] Rosandher Å. (2009). Olfactory Enrichment for Captive Snow Leopards (*Uncia uncia*).

[185] Farrell L.E., Roman J., Sunquist M.E. (2000). Dietary separation of sympatric carnivores identified by molecular analysis of scats. Mol. Ecol..

[186] Christiansen P., Harris J.M. (2012). Variation in craniomandibular morphology and sexual dimorphism in pantherines and the sabercat *Smilodon fatalis*. PLoS One.

[187] Kellert S.R., Black M., Rush C.R., Bath A.J. (1996). Human culture and large carnivore conservation in North America. Conserv. Biol..

[188] McCarthy K.P., Fuller T.K., Ming M., McCarthy T.M., Waits L., Jumabaev K. (2008). Assessing estimators of snow leopard abundance. J. Wildl. Manage..

[189] Wolf M., Ale S.O.M. (2009). Signs at the top: Habitat features influencing snow leopard *Uncia uncia* activity in Sagarmatha National Park, Nepal. J. Mammal..

[190] Marnewick K.A., Bothma J.d.P., Verdoorn G.H. (2006). Using camera-trapping to investigate the use of a tree as a scent-marking post by cheetahs in the Thabazimbi district. S. Afr. J. Widl. Res..

[191] Johnson R.P. (1973). Scent marking in mammals. Anim. Behav..

[192] Bothma J., Coertze R. (2004). Scent-marking frequency in southern Kalahari leopards. S. Afri. J. Wildl. Res..

[193] Jenny D. (1996). Spatial organization of leopards *Panthera pardus* in Taï National Park, Ivory Coast: Is rainforest habitat a ‘tropical haven’?. J. Zool..

[194] Aryal A., Kriegenhoffer B. (2009). Summer diet composition of the common leopard *Panthera pardus* (Carnivora: Felidae) in Nepal. J. Threaten. Taxa.

[195] Macri A.M., Patterson-Kane E. (2011). Behavioural analysis of solitary versus socially housed snow leopards (*Panthera uncia*), with the provision of simulated social contact. Appl. Anim. Behav. Sci..

[196] Visser R.R.C. (2002). Chemical Communication: Chemical Characterization of Volatile Constituents of Urine of the southern African Cheetah, Acinonyx Jubatus Jubatus, using Headspace Sampling and GC-MS.

[197] Reiger I. (1979). Scent rubbing in carnivores. Carnivora.

[198] Vaglio S., Minicozzi P., Bonometti E., Mello G., Chiarelli B. (2009). Volatile signals during pregnancy: A possible chemical basis for mother–infant recognition. J. Chem. Ecol..

[199] Smith T.E., Tomlinson A.J., Mlotkiewicz J.A., Abbott D.H. (2001). Female marmoset monkeys (*Callithrix jacchus*) can be identified from the chemical composition of their scent marks. Chem. Senses.

[200] Sonnet P.E. (1984). Gas chromatographic resolution and elution orders of simple diastereomeric alkenes. J. Chromatogr. A.

[201] Brahmachary R.L., Dutta J. (1981). On the Pheromones of Tigers: Experiments and Theory. Am. Nat..

[202] Wakte K.V., Thengane R.J., Jawali N., Nadaf A.B. (2010). Optimization of HS-SPME conditions for quantification of 2-acetyl-1-pyrroline and study of other volatiles in Pandanus amaryllifolius Roxb. Food Chem..

[203] Beaver B.V. (2009). CHAPTER 2-Canine Behavior of Sensory and Neural Origin. Canine Behavior.

[204] Beaver B.V. (2003). Chapter 1-Introduction to Feline Behavior. Feline Behavior.

[205] Soso S., Poddar-Sarkar M., Koziel J. Determining an optimal method of detection of odorous volatile organic compounds in tiger marking fluid in an effort to aid conservation.

[206] De Boer J.N. (1977). The age of olfactory cues functioning in chemocommunication among male domestic cats. Behav. Process..

[207] Natoli E. (1985). Behavioural Responses of Urban Feral Cats to Different Types of Urine Marks. Behaviour.

[208] Pageat P., Johnston D., Waner T. (1996). Functions and Uses of the Facial Pheromones in the Treatment of Urine Marking in the Cat.

